# An Efficacious Multi-Objective Fuzzy Linear Programming Approach for Optimal Power Flow Considering Distributed Generation

**DOI:** 10.1371/journal.pone.0149589

**Published:** 2016-03-08

**Authors:** Warid Warid, Hashim Hizam, Norman Mariun, Noor Izzri Abdul-Wahab

**Affiliations:** 1 Department of Electrical and Electronic Engineering, Faculty of Engineering, Universiti Putra Malaysia, 43400, UPM Serdang, Selangor, Malaysia; 2 Technical Institute Shatra, Southern Technical University, Foundation of Technical Education, Ministry of Higher Education & Scientific Research, Basrah, Iraq; 3 Centre for Advanced Power and Energy Research, Faculty of Engineering, Universiti Putra Malaysia, 43400, UPM Serdang, Selangor, Malaysia; Chongqing University, CHINA

## Abstract

This paper proposes a new formulation for the multi-objective optimal power flow (MOOPF) problem for meshed power networks considering distributed generation. An efficacious multi-objective fuzzy linear programming optimization (MFLP) algorithm is proposed to solve the aforementioned problem with and without considering the distributed generation (DG) effect. A variant combination of objectives is considered for simultaneous optimization, including power loss, voltage stability, and shunt capacitors MVAR reserve. Fuzzy membership functions for these objectives are designed with extreme targets, whereas the inequality constraints are treated as hard constraints. The multi-objective fuzzy optimal power flow (OPF) formulation was converted into a crisp OPF in a successive linear programming (SLP) framework and solved using an efficient interior point method (IPM). To test the efficacy of the proposed approach, simulations are performed on the IEEE 30-busand IEEE 118-bus test systems. The MFLP optimization is solved for several optimization cases. The obtained results are compared with those presented in the literature. A unique solution with a high satisfaction for the assigned targets is gained. Results demonstrate the effectiveness of the proposed MFLP technique in terms of solution optimality and rapid convergence. Moreover, the results indicate that using the optimal DG location with the MFLP algorithm provides the solution with the highest quality.

## 1 Introduction

Over the last decade, multi-objective optimal power flow (MOOPF) solution has gained considerable interest in power utilities because many real-world power system operation issues involve the simultaneous optimization of multiple, competing, and incommensurable objectives [1,2]. This solution is widely considered as an essential tool for system operators to maintain an economical, secure, and reliable operation of modern power systems [3]. At present, interrelated issues, including insufficient reactive power reserve margin, electricity market restructuring, and the increasing exploitation of distributed generation (DG), affect the operation strategies of many meshed power networks. Thus, power system decision makers have to make crucial decisions regarding unfamiliar groups of conflicting objectives. These new conditions have raised the need to develop realistic schemes for the MOOPF solution that incorporate new combinations of objective functions, planner past experiences, additional constraints, an expanded variety of system control variables, as well as DG effect.

As a sign, with the increased exploitation of power systems, frequent blackouts have been recorded worldwide [4]. In many cases, these circumstances have been attributed to insufficient MVAR reserve that results in voltage instability problems [5]. Furthermore, reforming the power sector with rigid economic restrictions, in which systems are operated under stress, also leads to the same pattern of incidents because of inadequate reactive power and voltage support with increased transmission losses [6]. Concurrently, a widespread increase in the penetration of DG technologies has been observed in large interconnected power systems [7]. At present, DG technologies offer economical and technical, benefits such as transmission cost minimization, congestion mitigation, and loss reduction, which can alleviate many harmful effects of the previously mentioned conditions [7,8].

A MOOPF problem is commonly modeled as a large-scale, nonlinear optimization problem with various operational constraints. In recent years, diverse heuristic optimization algorithms have been proposed and employed to solve different MOOPF problems, such as the Pareto-based multi-objective evolutionary algorithms (MOEA) [9], two-layer simulated annealing (TLSA) [10], the differential evolution (DE) algorithm [11], particle swarm optimization (PSO) [1], the enhanced genetic algorithm (EGA) [12], teaching learning-based optimization (TLBO) [13], and the artificial bee colony algorithm (ABC) [2,3].

Despite their theoretical features, these stochastic algorithms exhibit some drawbacks that confine their utilization in MOOPF software for practical power system operation [14,15]. The most serious and known cons of these methods are their algorithmic complexity [16], long computation time [15,17,18], insecure convergence and stagnation problem [2,16,19], and deficiencies in enforcing constraints [17].

In fact, fuzzy set optimization models offer compromise tools that perform such tasks. Using these tools, we can obtain realistic models that deal with given fuzzy objectives based the subjective targets and past experiences of a power system planner [20,21]. The viability of fuzzy set theory in various applications, including power system operation, planning, and control, has been proven; hence, this theory has been receiving increasing attention and has become the choice of many of electrical engineers [22]. In particular, fuzzy set theory is efficacious in solving MOOPF optimization problems [21]. This feature is attributed to its capability to coordinate various competence and incommensurable objectives of the optimization problem and provide a unique solution that successfully achieves the objectives of system planners. Consequently, a decision-making approach is not required to identify a compromise solution. Moreover, when fuzzy set theory is properly modeled, a considerable reduction in computational time can be achieved, which makes this approach applicable to real-time power system operations [20]. In recent years, only a few studies have documented the solution to the MOOPF problem using fuzzy logic approaches. El Sehiemy et al. [21] solved the MOOPF problem using a fuzzy-based technique by considering the minimization of reactive power generation cost and real power loss as objective functions. Abou El-Elaet al.[23] developed a modified multi-objective fuzzy linear programming optimization (MFLP) algorithm to solve the MOOPF problem by considering generation cost, preventive control action, as well as pre- and post-emergency conditions. Another application of fuzzy logic to the optimum real power dispatch problem, which considered preventive control action constraints, was presented in [24]. MFLP procedures were suggested for multi-objective optimal reactive power optimization [25,26]. Real power losses minimization and voltage stability margin maximization were selected as problem targets in [25]. The cost minimization of new switchable reactive power sources and real power losses were considered as problem objectives in [26]. Lastly, a fuzzy system was used to solve the reactive power control problem in hybrid electrical power systems in [20]. Notably, linear programming formulations have been used to formulate the mathematical framework for the aforementioned fuzzy set approaches because these formulations are fast, reliable, and viable tools for solving power grid online optimization problems that efficiently enforces inequality constraints [27,28].

Considering the aforementioned circumstances occurring in electricity markets and the growing role of decision makers in power systems operations, developing an efficient optimization technique that can solve new models of the MOOFP problem and satisfy the preferences of decision makers, is critical.

A considerable contribution can be made in this field in terms of enhancing exploration capability, modeling new combinations of optimal power flow (OPF) objectives, representing operational constraints, as well as studying the appropriate employing of DG technologies to achieve a better solution to the MOOPF problem.

In this study, an MFLP algorithm that deals with recent substantial developments is proposed to solve a new model of the MOOPF problem that considers a variant combination of objectives with and without optimum DG penetration. In the proposed MFLP algorithm, an extended group of control variables is used. The group comprises real power generation outputs, voltages of generation buses, tap setting of regulating transformers, reactive power of shunt capacitors, as well as active power generation of DG units. Three competing objectives that address these recent conditions are considered for the individual and simultaneous optimization process in this research, namely, minimization of real power loss, maximization of voltage stability margin, and maximization of the MVAR reserve margin of switchable sources. These objectives are fuzzified with suitable membership functions and extreme targets. The system constraints are regarded as hard constraints to maximize exploration capability and satisfy problem objectives. A successive linear programming (SLP) framework is employed to solve the proposed fuzzy approach using the interior-point method (IPM). The suggested MFLP approach coordinate objective functions and enforce the operational constraint strictly to provide the optimal setting of the control variables that achieve a single optimum solution. The IEEE 30-bus and IEEE 118-bus test systems are considered to demonstrate the efficacy of the suggested MFLP optimization algorithm. Different combinations of the three objectives with and without DG effect are solved. The rest of this article is organized as follows. Section 2 expresses the developed mathematical formulation of the MOOPF problem considering DG units parameters. In Section 3, the proposed MFLP optimization technique for solving the MOOPF problem with optimum DG penetration is presented. The simulation results, discussions, and comparisons with approaches reported in the literature are presented and described in Section 4. Lastly, conclusions are drawn in Section 5.

## 2 Problem Formulation

Basically, multi-objective optimization problems involve minimizing and/or maximizing a set of contradictory objective functions subject to a wide variety of constraints. A single optimum solution with tradeoffs between two or more competing goals is needed [29,30]. This study aims to estimate the optimum adjustment of the control variables in terms of the considered objectives meanwhile achieving a group of equality and inequality constraints. Moreover, DG optimum sizing is considered another objective to obtain a more optimal solution to the MOOPF problem. Thus, DG size is added to the set of control variables.

Table 1 lists the symbols and abbreviations used throughout this paper.

**Table 1:** List of symbols and abbreviations.

Notations	Description
*F*_*jk*_	A submatrix that is obtained from *Y* bus matrix.
*I*_*line*,*i*_	Current magnitude of the *i*th transmission line (kA).
$I_{line,i}^{\text{max}}$	Current magnitude limit of the *i*th transmission line (kA).
*Jac*	Jacobian matrix.
*L*_*j*_	L-index value of the *j*th load bus.
*N*_*c*_	Number of compensation capacitors.
*N*_*DG*_	Number of DG units.
*N*_*G*_	Number of generation units.
*N*_*L*_	Number of load buses.
*N*_*l*_	Number of transmission lines.
*N*_*T*_	Number of regulating transformers.
*P*_*DGi*_	Active power generation of the *i*th DG unit (MW).
$P_{DGi}{}^{\text{max}}$	Maximum real power output of the *i*th DG unit (MW).
${P_{DGi}}^{\text{min}}$	Minimum real power output of the *i*th DG unit (MW).
*P*_*Di*_	Active power demand at the *i*th bus (MW).
*P*_G1_	Swing bus active power (MW).
$P_{G1}{}^{\text{max}}$	Maximum real power output of the swing bus (MW).
$P_{G1}{}^{\text{min}}$	Minimum real power output of the swing bus (MW).
*P*_*Gi*_	Real power output of the *i*th generation unit (MW).
$P_{Gi}{}^{\text{max}}$	Maximum real power output of the *i*th generation unit (MW).
$P_{Gi}{}^{\text{min}}$	Minimum real power output of the *i*th generation unit (MW).
*P*_*loss*_	Active power loss (MW).
*QC*_*RM*_	MVAR reserve margin of compensator capacitors.
$Q_{Capi}{}^{\text{min}}$	Lower reactive power limit of the *i*th compensator capacitor (MVAr).
*Q*_*DGi*_	Reactive power generation of *i*th DG unit (MVAr).
$Q_{DGi}{}^{\text{max}}$	Maximum reactive power output of the *i*th DG unit (MVAr).
$Q_{DGi}{}^{\text{min}}$	Minimum reactive power output of the *i*th DG unit (MW).
*Q*_*Di*_	Reactive power demand at the *i*th bus (MVAr).
*Q*_*Gi*_	Reactive power of *i*th generator bus (MVAr).
$Q_{Gi}{}^{\text{max}}$	Maximum reactive power output of the *i*th generation unit (MVAr).
$Q_{Gi}{}^{\text{min}}$	Minimum reactive power output of the *i*th generation unit (MW).
*Tap*_*i*_	Tap setting of the *i*th regulating transformer.
$Tap_{i}{}^{\text{max}}$	Maximum tap setting limit of the *i*th regulating transformer.
$Tap_{i}{}^{\text{min}}$	Minimum tap setting limit of the *i*th regulating transformer.
*u*	Modified version vector of control variables.
VSEI	Voltage stability enhancement index.
*V*_*Gi*_	Voltage amplitude of the *i*th generation bus (p.u.).
$V_{Gi}{}^{\text{max}}$	Maximum limit for the voltage magnitude of the *i*th generator (p.u.).
$V_{Gi}{}^{\text{min}}$	Minimum limit for the voltage magnitude of the *i*th generator (p.u.).
*V*_*Li*_	Voltage amplitude of the*i*th load bus (p.u.).
$V_{Li}{}^{\text{max}}$	Maximum limit for the voltage magnitude of the *i*th load bus (p.u.).
$V_{Li}{}^{\text{min}}$	Minimum limit for the voltage magnitude of the *i*th load bus (p.u.).
*x*	Modified version vector of dependent variables.
*Y*	Bus admittance matrix.
*μ*(*P*_*loss*_)	Fuzzy membership function for active power losses.
*μ*(*QC*_*RM*_)	Fuzzy membership function for switchable VAR sources reserve margin maximization.
*μ*(VSEI)	Fuzzy membership function for VSEI.
*λ*	Satisfaction factor.
$\frac{\partial P_{loss}}{\partial P_{Gi}}$	Sensitivity of real power loss to real power output of the *i*th generation unit.
$\frac{\partial P_{loss}}{\partial P_{DGi}}$	Sensitivity of real power loss to active power generation of the *i*th DG unit.
$\frac{\partial P_{loss}}{\partial Q_{capi}}$	Sensitivity of real power loss to reactive power output of the *i*th compensator capacitor at load bus *i*.
$\frac{\partial P_{loss}}{\partial V_{Gi}}$	Sensitivity of real power loss to voltage magnitude for the *i*th generation bus.
$\frac{\partial P_{loss}}{\partial Tap_{i}}$	Sensitivity of real power loss to tap setting of the *i*th regulating transformer.
$\frac{\partial QC_{RM}}{\partial Q_{capi}}$	Sensitivity of MVAR reserve margin of compensator capacitors to reactive power output of the *i*th compensator capacitor.
$\frac{\partial \text{VSEI}}{\partial V_{Gi}}$	Sensitivity ofvoltage stability enhancement index to voltage magnitude of the *i*th generation bus.
Δ*P*_*DGi*_	Incremental active power generation of the *i*th DG unit.
Δ*P*_*Gi*_	Incremental real power output of the *i*th generation unit.
Δ*Q*_*capi*_	Incremental reactive power output of the *i*th compensator capacitor.
Δ*Tap*_*i*_	Incremental tap setting of the *i*th regulating transformer.
Δ*u*	Optimum increment of the independent variables.
Δ*V*_*Gi*_	Incremental voltage amplitude of the *i*th generation bus.
Δ*λ*	Optimum increment of the membership satisfaction.

The general mathematical formulation of MOOPF problem is stated as follows:

$$\text{Minimize }f_{i}(x,u)\;i=1,2,\ldots ,N_{obj}$$ (1)

Subjected to:

$$g_{i}(x,u)=0\;i=1,2,\ldots ,J$$ (2)

$$h_{i}(x,u)\leq 0\;i=1,2,\ldots ,H$$ (3)

where *f*_*i*_ is the *i*th objective function, *N*_*obj*_ is the number of objectives, *g*_*i*_(*x*,*u*) is the *i*th equality constraint, and *h*_*i*_(*x*,*u*) is the *i*th inequality constraint. *J* and *H* are the number of equality constraints that represent the power flow equations and the number of inequality constraints, respectively. In this paper, we have added DG reactive power generation *Q*_*DG*_ to the dependent variables. The modified version vector of dependent variables can be expressed as:

$$x={\left[P_{G1},\;Q_{G1},\ldots ,Q_{GN_{G}},\;Q_{DG1},\ldots ,Q_{DGN_{DG}},\;V_{L1},\ldots ,V_{LN_{L}},\;I_{line,1},\ldots ,I_{line,N_{l}}\right]}^{T}$$ (4)

In addition, we have added DG real power generation to the control variables. The modified version vector of the control variables consists of real power generation outputs except for slack bus, active power generation of DG units *P*_*DG*_, reactive power outputs of compensator capacitors *Q*_*cap*_, voltages of generation buses *V*_*G*_, and tap setting of regulating transformers units *Tap*. Hence, *u* can be represented as:

$$u={\left[P_{G2},\ldots ,P_{GN_{G}},\;P_{DG1},\ldots ,P_{DGN_{DG}},\;Q_{cap1},...,Q_{capN_{C}},\;V_{G1},...,V_{GN_{G}},\;Tap_{1},\ldots ,Tap_{N_{T}}\right]}^{T}$$ (5)

### 2.1 Problem objective functions

Based on power utilities selection as well as previously mentioned justification in Section 1 that explains the recent substantial development occurring in the electricity market, three conflicting objectives are considered in the present study to solve the MOOPF problem. These objectives are: real power loss minimization, switchable MVAR sources reserve margin maximization, and voltage stability enhancement maximization. Furthermore, the maximization saving by using DG is implicitly considered as an additional optimization objective in the proposed formulation. A sensitivity-based formula is proposed to identify the candidate location(s) for DG units placement. Meanwhile, DG optimal size is treated as an additional control variable to optimally adjust in the way that produces maximum saving in total cost of power generation. The general mathematical formulation of MOOPF problem that considers maximization of the saving by using DG, which are given in Eqs (1–3), can be expressed as follows:-

$$\text{Minimize }[P_{loss},\text{ VSEI}]$$ (6)

$$\text{Maximize }[QC_{RM}]$$ (7)

Subjected to:

$$g_{i}(x,u)=0\;i=1,2,\ldots ,J$$ (8)

$$h_{i}(x,u)\leq 0\;i=1,2,\ldots ,H$$ (9)

Detailed explanation which justifies the selection of each considered objective function is given below.

#### 2.1.1 Minimization of real power loss

The minimization of active power loss *P*_*loss*_ is a widely used objective for the optimal power flow problem [28,31]. Furthermore, it is an essential issue that is frequently considered to enhance power delivery. Thus, minimizing active power loss is the main objective that should be achieved optimally in our work. The real power loss of a meshed power system can be estimated as follows:

$$P_{loss}=\sum_{i=1}^{N}\sum_{\begin{array}{c} j=1\\ j\neq i \end{array}}^{N}\begin{array}{cc} \frac{G_{ij}}{2} & \left[{\left|\;V_{i}\right|}^{2}+{\left|\;V_{j}\right|}^{2}-2|\;V_{i}||\;V_{j}|cos(\delta_{i}-\delta_{j})\right] \end{array}$$ (10)

where *N* is the total number of power system buses, *G*_*ij*_ is the conductance of a transmission line that joins the *i*th and *j*th buses, *V*_*i*_ is the voltage magnitude of the *i*th bus, *V*_*j*_ is the voltage magnitude of the *j*th bus, and *δ*_*i*_ and *δ*_*j*_ are the bus voltage angles at the terminals of the *i*–*j* transmission line.

#### 2.1.2 Switchable MVAR sources reserve margin maximization

With the recent growth of loading rates in existing power systems worldwide, the prominence of maximizing the MVAR reserve margin of compensator capacitors has steadily increased. This important goal to satisfy in term of supplying the desired reactive power throughout contingency cases. This objective can be formulated as follows:

$$\text{max }QC_{RM}=\sum_{i=1}^{N_{c}}\left(Q_{capi}{}^{\text{max}}-Q_{capi}\right)$$ (11)

where *Q*_*capi*_ is the reactive power output of the *i*th compensator capacitor. Meanwhile, $Q_{capi}{}^{\text{max}}$ is the upper reactive power limit of the *i*th compensator capacitor.

#### 2.1.3 Voltage stability enhancement

Considering voltage stability has become an essential concern in the optimal power flow problem because of the growth of transmission system loading that leads to numerous voltage collapse incidences. To assess the voltage stability of a specified power network, an efficient indicator of the load bus L-index [32] is widely used with consistent results. By employing normal load flow data, the L-index produces a scalar value that varies from 0 (no load) to 1 (voltage collapse). The L-index value indicates the proximity of each load bus to voltage collapse; hence, it determines weak load buses that require MVAR support. The L-index of the *k*th load bus can be written as follows:

$$L_{k}=|1-\sum_{j=1}^{N_{G}}F_{jk}\frac{V_{Gj}}{V_{Lk}}|\text{ k}=1,2,\ldots ,N_{L}$$ (12)

where *V*_*Gj*_ is the voltage of the *j*th generation bus and *V*_*Lk*_ is the voltage of the *k*th load bus. The values of *F*_*jk*_ are fixed for a specific power system configuration which can be computed from the *Y* bus matrix. Consequently, to preserve voltage stability, the objective should be to minimize the summation of square L-indexes for a given load case, which can be called a voltage stability enhancement index. This index is expressed as follows:

$$\text{Min VSEI}=\sum_{k=1}^{N_{L}}{\text{L}}_{k}^{2}$$ (13)

Moreover, a global power network *L*-index indicating the proximity of the network to voltage collapse can be defined as the maximum of *L*-indices, i.e.,*L*_max_. A low *L*_max_ value indicates a high degree of voltage stability.

### 2.2 Problem constraints

The optimization process involves two main groups of constraints that should be maintained within their limits. These restrictions can be categorized as follows.

#### 2.2.1 Equality constraints

The equality constraints describe load flow equations that control the power system; they are generally called real and reactive power balance. These constraints can be expressed as follows:

$$P_{Gi}-P_{Di}=V_{i}\sum_{j=1}^{N}V_{j}(G_{ij}cos\delta_{ij}+B_{ij}sin\delta_{ij})$$ (14)

$$Q_{Gi}-Q_{Di}=V_{i}\sum_{j=1}^{N}V_{j}(G_{ij}sin\delta_{ij}-B_{ij}cos\delta_{ij})$$ (15)

where *B*_*ij*_ is the susceptance of a transmission line that joins the *i*th and *j*th buses, where as *δ_ij_* = *δ_i_* − *δ_j_* is the voltage angle difference between the *i*th and *j*th nodes.

#### 2.2.2 Inequality constraints

In formulating the optimal power flow problem, a group of inequality constraints are considered. These constraints can be called power system operating constraints, which include the limits of physical devices and the borders formed to maintain system security. In this research, for consistency with the proposed approach, we classify the inequality constraints based on the type of variable; as control variables constraints that belong to *u* vector Eq (5) and dependent variables constraints which stated in *x* vector Eq (4). The control variables constraints can be expressed as follows:

$$P_{Gi}{}^{\text{min}}\leq P_{Gi}\leq P_{Gi}{}^{\text{max}}\;i=1,2,\ldots ,N_{G}-1$$ (16)

$$P_{DGi}{}^{\text{min}}\leq P_{DGi}\leq P_{DGi}{}^{\text{max}}\;i=1,2,\ldots ,N_{DG}$$ (17)

$$Q_{capi}{}^{\text{min}}\leq Q_{capi}\leq Q_{capi}{}^{\text{max}}\;i=1,2,\ldots ,N_{c}$$ (18)

$$V_{Gi}{}^{\text{min}}\leq V_{Gi}\leq V_{Gi}{}^{\text{max}}\;i=1,2,\ldots ,N_{G}$$ (19)

$$Tap_{i}{}^{\text{min}}\leq Tap_{i}\leq Tap_{i}{}^{\text{max}}\;i=1,2,\ldots ,N_{T}$$ (20)

While the dependent variables constraints can be described as follows:

$$P_{G1}{}^{\text{min}}\leq P_{G1}\leq P_{G1}{}^{\text{max}}$$ (21)

$$Q_{Gi}{}^{\text{min}}\leq Q_{Gi}\leq Q_{Gi}{}^{\text{max}}\;i=1,2,\ldots ,N_{G}$$ (22)

$$Q_{DGi}{}^{\text{min}}\leq Q_{DGi}\leq Q_{DGi}{}^{\text{max}}\;i=1,2,\ldots ,N_{DG}$$ (23)

$$V_{Li}{}^{\text{min}}\leq V_{Li}\leq V_{Li}{}^{\text{max}}\;i=1,2,\ldots ,N_{L}$$ (24)

$$I_{line,i}\leq I_{line,i}^{\text{max}}\;i=1,2,\ldots ,N_{l}$$ (25)

## 3 Proposed MFLP Algorithm for the MOOPF Problem Considering DG

In this section, a MFLP methodology is suggested to solve a new model of the MOOPF problem with and without considering the influence of DG to assess its effect on solution quality and convergence rapidity. This solution is achieved by performing simultaneous optimization for the three competing objective functions that address the aforementioned issues. The challenge is to solve this problem with high satisfaction while achieving optimal power system operation and enforcing security constraints. Hence, these objectives are fuzzified with appropriate membership functions that consider extreme targets. Notably, DG optimal placement is incorporated into the proposed MFLP. The aim is to achieve higher optimality by estimating the optimum capacity for DG.

### 3.1 Fuzzification of the objective functions

**Fig 1:**
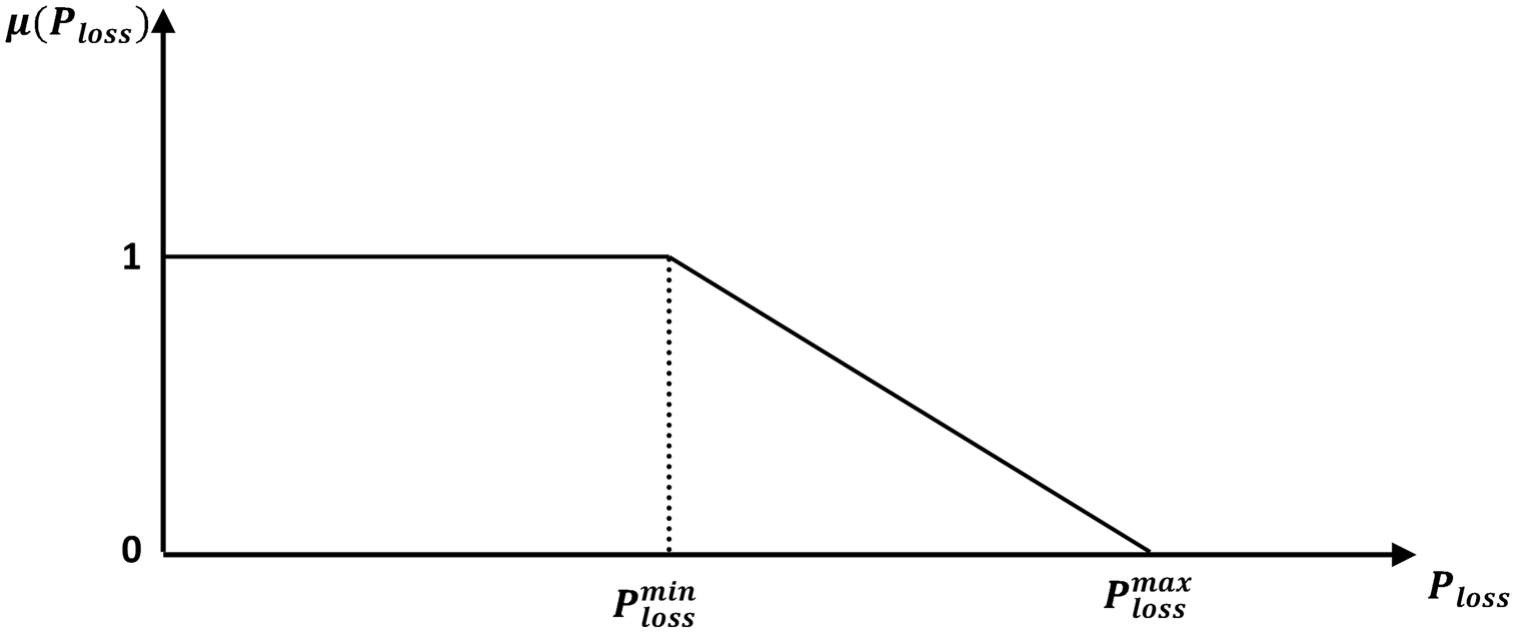
Fuzzy membership function for active power losses minimization.

The three nominated objective functions are characterized by their conflicting nature. A minimization membership function is depicted for each of the active power loss and the voltage stability index. Meanwhile, a maximization membership function is used for the shunt MVAR compensators reserve. The objective functions are transformed from crisp values mode into fuzzy membership grades and treated as fuzzy constraints. The membership function of active power loss shown in Fig 1 can be expressed as follows:

$$\mu (P_{loss})=\{\begin{array}{cc} 1 & P_{loss}\leq P_{loss}^{min}\\ \left(\frac{P_{loss}^{max}-P_{loss}}{P_{loss}^{max}-P_{loss}^{min}}\right) & P_{loss}^{min}\leq P_{loss}\leq P_{loss}^{max}\\ 0 & P_{loss}\geq P_{loss}^{max} \end{array}$$ (26)

where $P_{loss}^{min}$ is the target value for the real power loss prescribed by the decision maker based on experience and preference, whereas $P_{loss}^{max}$ represents the estimated loss value for the initial load flow solution state.

**Fig 2:**
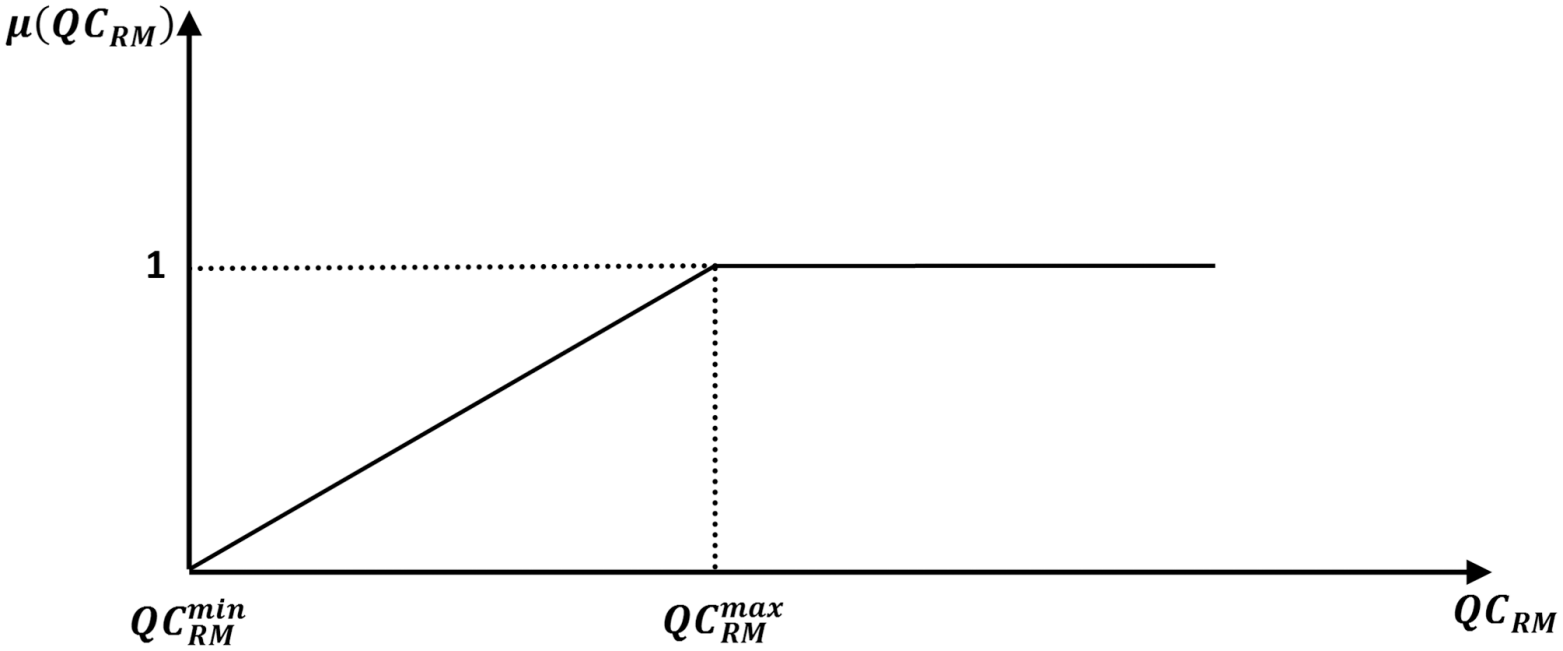
Fuzzy membership function for switchable VAR sources reserve margin maximization.

Moreover, a fuzzy membership function for the switchable MVAR reserve margin maximization can be depicted as shown in Fig 2. This function is given by

$$\mu (QC_{RM})=\{\begin{array}{cc} 1 & QC_{RM}\geq QC_{RM}^{max}\\ \left(\frac{QC_{RM}-QC_{RM}^{min}}{QC_{RM}^{max}-QC_{RM}^{min}}\right) & QC_{RM}^{min}\leq QC_{RM}\leq QC_{RM}^{max}\\ 0 & QC_{RM}\leq QC_{RM}^{min} \end{array}$$ (27)

where $QC_{RM}^{min}$&$QC_{RM}^{max}$ are the lower as well as the upper MVAR reserve margins for the compensator capacitors that are connected to selected load buses, respectively. Similarly, specifying the values of these parameters depends on the operational experiences of the decision makers.

**Fig 3:**
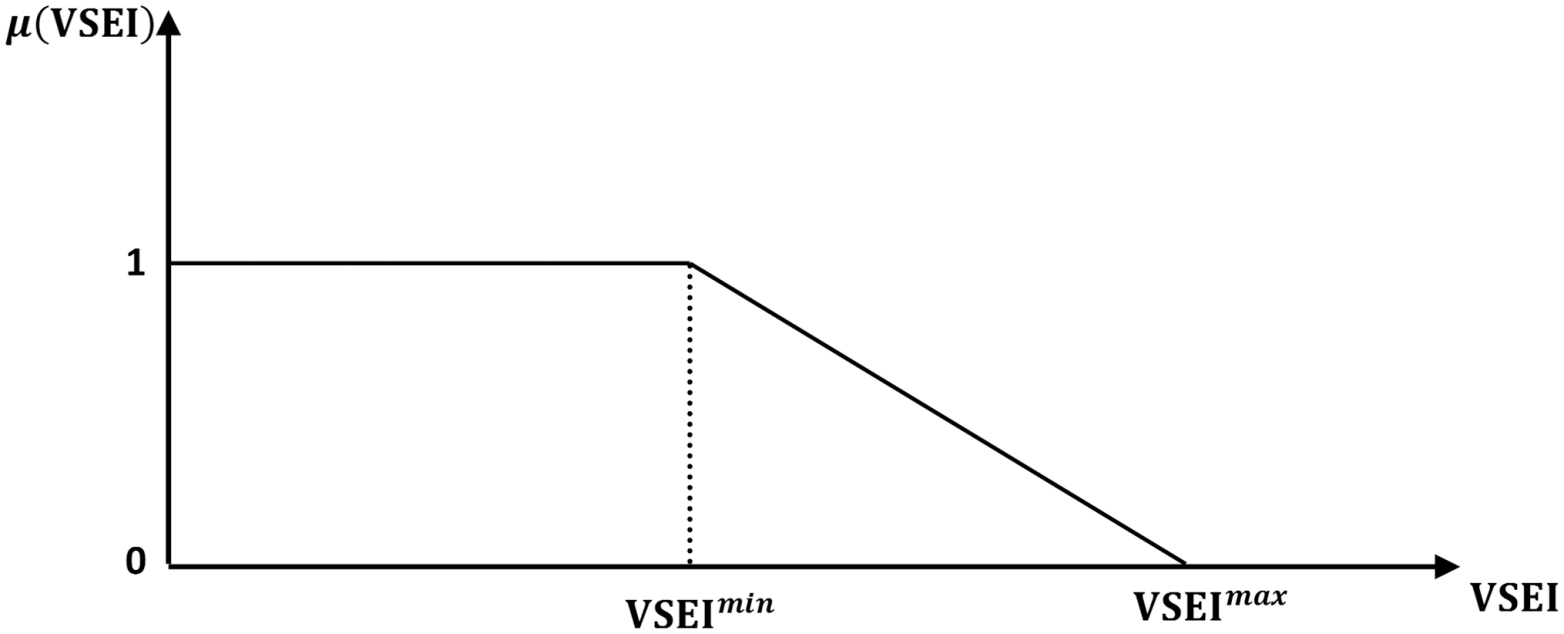
Fuzzy membership function for VSEI.

Meanwhile, Fig 3 describes the fuzzy membership function of the VSEI that can be expressed as follows:

$$\mu (\text{VSEI})=\{\begin{array}{cc} 1 & \text{VSEI}\leq {\text{VSEI}}^{min}\\ \left(\frac{{\text{VSEI}}^{max}-\text{VSEI}}{{\text{VSEI}}^{max}-{\text{VSEI}}^{min}}\right) & {\text{VSEI}}^{min}\leq \text{VSEI}\leq {\text{VSEI}}^{max}\\ 0 & \text{VSEI}\geq {\text{VSEI}}^{max} \end{array}$$ (28)

where VSEI^max^ is the computed voltage stability index for the present operating state, whereas VSEI^min^ is the desired value for the voltage stability index that can be subjectively assigned.

### 3.2 MFLP optimization model

The basic concept of the suggested methodology is to transform the fuzzy modeling of the MOOPF problem into a crisp model, and thus, obtain an optimal solution that simultaneously optimizes the objectives and strictly enforces all the constraints. In this research, we have treated all the constraints of the control and dependent variables as crisp constraints. This option is suitable when obtaining a high satisfaction for the objective functions by minimizing the number of membership functions during the optimization process is a priority. A satisfaction factor *λ* represents the optimality degree that is defined as the minimum of all the fuzzy membership functions of the problem objectives is proposed. Based on this scenario, the satisfaction factor formula can be expressed as follows:

$$\lambda =min[\mu (P_{loss}),\mu (QC_{RM}),\mu (\text{VSEI})]$$ (29)

where *λ*, *μ*(*P*_*loss*_), *μ*(*QC*_*RM*_), and *μ*(VSEI) are within the range of [0–1]. The goal is to maximize *λ*, that is, to maximize the values of the membership functions for the problem objectives symmetrically. A high fuzzy membership function value indicates a high degree of optimality.

Based on the previous statement, the problem of the proposed MFLP approach will be to maximize satisfaction factor. Considering the problem objectives as fuzzy constraints and the system constraints as hard constraints, the general mathematical formulation of MOOPF that considers DG, which are provided in Eqs (6–9), can be expressed based on Eq (29) as a semi-fuzzy optimal power flow optimization problem with hard constraints as shown below:-

Maximize λ (30)

Subject to

$$\lambda \leq \mu (P_{loss})$$ (31)

$$\lambda \leq \mu (QC_{RM})$$ (32)

λ≤μ(VSEI) (33)

0≤λ≤1 (34)

$$g_{i}(x,u)=0\;i=1,2,\ldots ,J$$ (35)

$$h_{i}(x,u)\leq 0\;i=1,2,\ldots ,H$$ (36)

This MOOPF problem can be converted into an entirely crisp problem by substituting the membership functions of the objective functions Eqs (26–28) into Eqs (31–33), respectively. Thus, the problem objectives can be treated as crisp constraints. Moreover, the aforementioned objective can be the minimization of −*λ*, which is mathematically equivalent to the maximization of *λ*. An elaborate form of the proposed MFLP model that considers DG can be expressed in terms of problem objectives, control variables, and dependent variables as follows:

min -λ (37)

Subjected to

(a) Fuzzy objective functions as crisp constraints.

$$\text{Min }(P_{loss}):\;P_{loss}+(P_{loss}^{max}-P_{loss}^{min})\lambda \leq P_{loss}^{max}$$ (38)

$$\text{Max }(QC_{RM}):\;-QC_{RM}+(QC_{RM}^{max}-QC_{RM}^{min})\lambda \leq -QC_{RM}^{min}$$ (39)

$$\text{Min }(\text{VSEI}):\text{ VSEI}+({\text{VSEI}}^{max}-{\text{VSEI}}^{min})\lambda \leq {\text{VSEI}}^{max}$$ (40)

(b) Control variables crisp constraints that consider DG as stated in Eqs (16–20).
(c) Dependent variables crisp constraints that consider DG as shown in Eqs (21–25).
(d) Satisfaction factor limits.

0≤λ≤1 (41)

The preceding multi-objective optimization problem can be solved to maximize the satisfaction factor while strictly enforcing the operational constraints by using an efficient SLP technique powered by an efficient interior point method solver.

### 3.3 Solution procedure for the proposed MFLP approach

**Fig 4:**
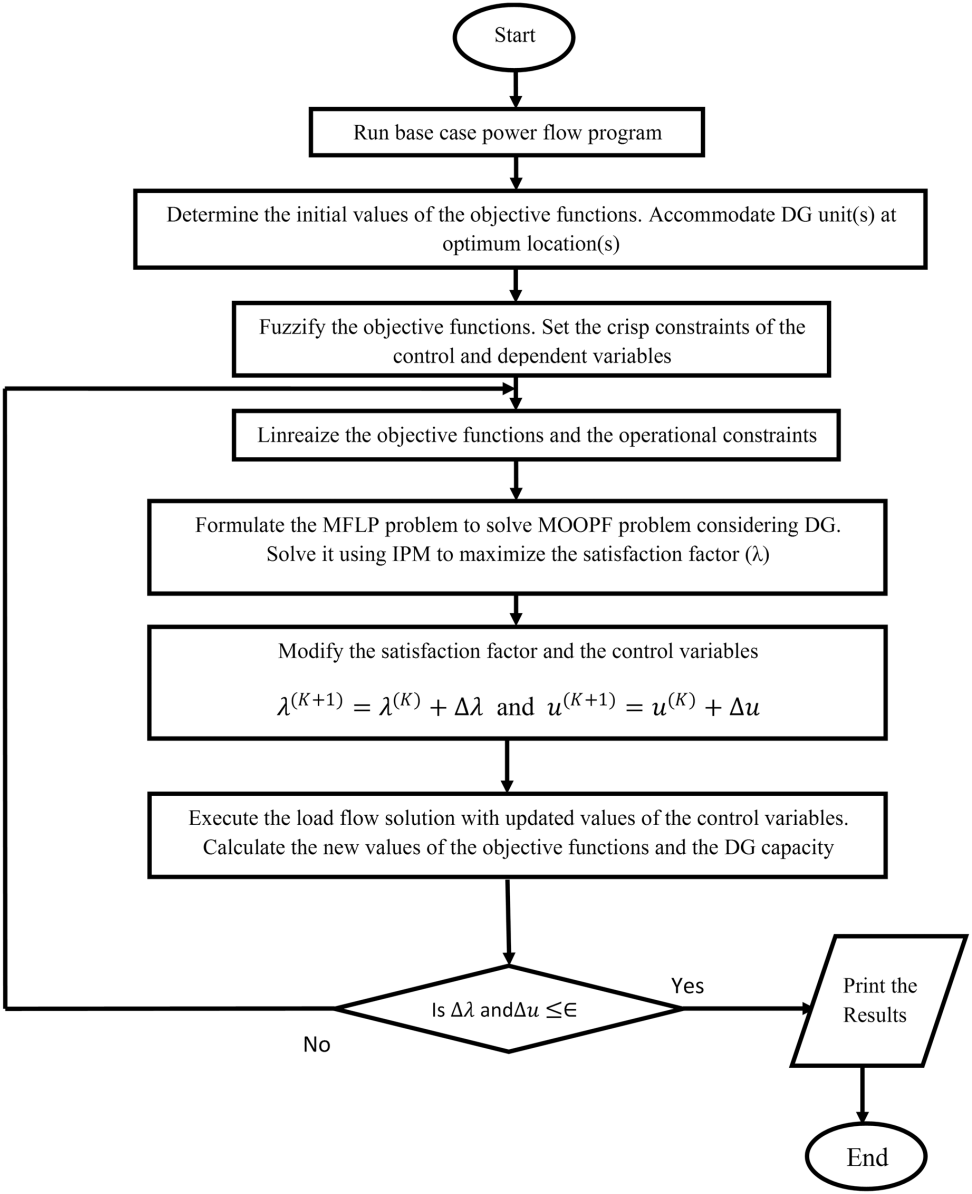
Flowchart of the proposed MFLP algorithm for the MOOPF problem considering DG.

The basic steps of the proposed MFLP approach to solve the MOOPF problem that considers DG are outlined below and illustrated in the flowchart presented in Fig 4.

Step 1 Solve the base case power flow. Estimate the initial values of the objective functions for the current operating state, which include real power loss minimization, shunt capacitors reserve margin maximization, and voltage stability index maximization using Eqs (10), (11), and (13), respectively.
Step 2 Accommodate the DG unit(s) at the candidate location(s) based on the sensitivity of real power losses to both of the injected real and reactive power as shown in the following matrix formula:

$$\begin{array}{l} \left[\begin{array}{c} \frac{\partial P_{loss}}{\partial P_{i}}\\ \frac{\partial P_{loss}}{\partial Q_{K}} \end{array}\right]={\left[Jac\right]}^{T^{-1}}\left[\begin{array}{c} \frac{\partial P_{loss}}{\partial \delta_{i}}\\ \frac{\partial P_{loss}}{\partial V_{K}} \end{array}\right]\;\\ \text{ with }i=2,\ldots ,N\;and\;k=1,\ldots ,N_{L} \end{array}$$ (42)

where $\frac{\partial P_{loss}}{\partial P_{i}}$ is the change in real power loss to injected real power through *i*th bus, whereas $\frac{\partial P_{loss}}{\partial Q_{K}}$ represents the change in real power loss to injected reactive power through *k*th load bus. $\frac{\partial P_{loss}}{\partial \delta_{i}}$ is the sensitivity of real power loss to voltage angle for the ith bus. $\frac{\partial P_{loss}}{\partial V_{K}}$ is the sensitivity of real power loss to voltage magnitude for the kth load bus.

Step 3 Fuzzify the objective functions in Eqs (26–28) based on the initial state conditions and the assigned targets. Specify the control and dependent variables crisp constraints.
Step 4 Linearize the fuzzy objective functions in Eqs (38–40) and the crisp constraints in Eqs (16–25). The linearized constraints of the objective functions can be expressed as follows:

$$(P_{loss}^{max}-P_{loss}^{min})\Delta \lambda +\Delta P_{loss}\leq P_{loss}^{max}-P_{loss}^{\left(K\right)}-(P_{loss}^{max}-P_{loss}^{min})\lambda^{\left(K\right)}$$ (43)

$$(QC_{RM}^{max}-QC_{RM}^{min})\Delta \lambda -\Delta QC_{RM}\leq -QC_{RM}^{min}+QC_{RM}^{\left(K\right)}-(QC_{RM}^{max}-QC_{RM}^{min})\lambda^{\left(K\right)}$$ (44)

$$({\text{VSEI}}^{max}-{\text{VSEI}}^{min})\Delta \lambda +\Delta \text{VSEI}\leq {\text{VSEI}}^{max}-{\text{VSEI}}^{\left(\text{K}\right)}-({\text{VSEI}}^{max}-{\text{VSEI}}^{min})\lambda^{\left(K\right)}$$ (45)

where *K* denotes the iteration number.Δ*P*_*loss*_, Δ*QC*_*RM*_, and ΔVSEI are the incremental real power loss, the change in shunt compensators MVAR reserve, and the incremental voltage stability index, respectively. Each incremental component includes a sensitivity vector that describes the change in objective function to the control variables. These vectors can be formulated as follows:

$$\Delta P_{loss}=[\begin{array}{cc} \begin{array}{cc} \begin{array}{ccc} \frac{\partial P_{loss}}{\partial P_{Gi}} & \frac{\partial P_{loss}}{\partial P_{DGi}} & \frac{\partial P_{loss}}{\partial Q_{capi}} \end{array} & \frac{\partial P_{loss}}{\partial V_{Gi}} \end{array} & \frac{\partial P_{loss}}{\partial Tap_{i}} \end{array}][\begin{array}{c} \Delta P_{Gi}\\ \Delta P_{DGi}\\ \Delta Q_{capi}\\ \begin{array}{c} \Delta V_{Gi}\\ \Delta Tap_{i} \end{array} \end{array}]$$ (46)

$$\Delta QC_{RM}=[\begin{array}{c} \begin{array}{c} \begin{array}{ccc} \frac{\partial QC_{RM}}{\partial Q_{cap1}} & \frac{\partial QC_{RM}}{\partial Q_{cap2}}\ldots \ldots \ldots .. & \frac{\partial QC_{RM}}{\partial Q_{capN_{c}}} \end{array} \end{array} \end{array}][\begin{array}{c} \begin{array}{c} \Delta Q_{cap1}\\ \begin{array}{c} \Delta Q_{cap2}\\ \vdots \\ \vdots \\ \Delta Q_{capN_{c}} \end{array} \end{array} \end{array}]$$ (47)

$$\Delta \text{VSEI}=[\begin{array}{c} \begin{array}{c} \begin{array}{ccc} \frac{\partial \text{VSEI}}{\partial V_{G1}} & \frac{\partial \text{VSEI}}{\partial V_{G2}}\ldots \ldots \ldots .. & \frac{\partial \text{VSEI}}{\partial V_{GN_{G}}} \end{array} \end{array} \end{array}][\begin{array}{c} \begin{array}{c} \Delta V_{G1}\\ \begin{array}{c} \Delta V_{G2}\\ \vdots \\ \vdots \\ \Delta V_{GN_{G}} \end{array} \end{array} \end{array}]$$ (48)

Step 5 Formulate the proposed MFLP approach to achieve maximum satisfaction.
Step 6 Solve the MFLP problem using an interior point algorithm to determine the optimum increment of the independent variables Δ*u* and the membership satisfaction Δ*λ*. Update the values of the control variables vector *u* as *u*^(*K*+1)^ = *u*^(*K*)^+Δ*u*. Modify the satisfaction factor *λ* as *λ*^(*K*+1)^ = *λ*^(*K*)^+Δ*λ*. Where Δ*u* = [Δ*P*_*Gi*_ Δ*P*_*DGi*_ Δ*Q*_*capi*_ Δ*V*_*Gi*_ Δ*Tap*_*i*_]^*T*^.
Step 7 Perform load flow using the new values of the control variables. Determine the modified values of the objective functions and DG penetration.
Step 8 Check if Δ*λ* and Δ*u* are less than the assigned tolerance. Otherwise, return to Step 4 until the stopping criterion is achieved.

The above solution procedure can be used for single objective optimization cases. Thus, the proposed MFLP can be named as a fuzzy linear programming (FLP) algorithm.

## 4 Results and Discussions

To demonstrate the effectiveness of the suggested MFLP technique for the MOOPF problem considering DG, the procedure is applied in the IEEE 30-bus and IEEE 118-bus test systems. As stated previously, three objective functions are regarded to solve the MOOPF problem that considers DG, namely, real loss minimization, voltage stability enhancement, and shunt capacitors MVAR reserve maximization, using the suggested MFLP algorithm. From an economic perspective, certain load bus is suitable for accommodating a single DG unit with a high capacity instead of installing many DG units at several load buses. In this study and based on this concept, a single DG unit is used. A common type of DG unit that can generate real and reactive power is considered during the execution of the proposed MFLP algorithm with 10 MW maximum capacity and 0.85 p.f. Synchronous machines (e.g. reciprocating engines, cogeneration, combustion gas turbine, etc.) are under this category. To compare and assess the efficacy and robustness of the proposed MFLP algorithm with and without the DG effect, simulations are performed for several cases. In this work, the cases are divided into two categories. In the first category, the problem objectives are optimized individually. In the second category, the objectives are optimized simultaneously. To implement the proposed MFLP algorithm in these cases, the maximum and minimum values of the problem fuzzy objectives are required. The values of $P_{loss}^{max}$ and VSEI^max^ are obtained from the results of the base case power flow. Meanwhile, an extremely target values for the problem objectives $P_{loss}^{min}$,VSEI^min^, and $QC_{RM}^{max}$ are assigned to investigate the robustness of the proposed approach. In addition, three well-known algorithms are utilized as competitors to solve the multi-objective OPF problem. These algorithms include Non-dominated Sorting Genetic Algorithm-II (NSGA-II) [33], Neighborhood Knowledge-based Evolutionary Algorithm (NKEA) [34], and Differential Evolution (DE) [35]. The proposed work is implemented in the computational environment of MATLAB R2015a and executed on a PC with 2.4 GHz Intel^®^ Core™ i7 CPU and 8 GB RAM. The five considered cases of single and multi-objective optimization are

Case-1 Active power losses minimization.
Case-2 Voltage stability enhancement.
Case-3 Minimization of real power loss and maximization of shunt capacitors MVAR reserve.
Case-4 Minimization of real power loss and the voltage stability index.
Case-5 All objectives are optimized simultaneously.

### 4.1 IEEE 30-bus test system

The complete data of this system [36] comprise branch parameter, load data, generator data, and the initial setting of the control variables with their corresponding operational constraints. This network consists of 41 transmission lines, 6 generator buses, and 24 load buses. Four branches, namely, (6–9), (6–10), (4–12), and (27–28), are equipped with tap-changing transformers. Meanwhile, load buses 10, 12, 15, 17, 20, 21, 23, 24, and 29 are chosen as locations for the switchable shunt capacitors. Bus 1 is selected as the swing bus and generator buses 2, 5, 8, 11, and 13 are regarded as PV buses. On the one hand, the minimum and upmost boundaries of the voltage magnitude of the generation units and the load buses are set to [0.95, 1.1] p.u and [0.95, 1.05] p.u, respectively. On the other hand, the upper and lower limits for each transformer tap are 0.9 p.u and 1.1 p.u, respectively. The MVAR injected by each capacitor bank is within 0–5 MVAR. Table 2 presents the assigned parameters of the fuzzy membership functions for the problem objectives of the modified IEEE 30-bus test system.

**Table 2:** Parameters of the fuzzy membership functions for the problem objectives (IEEE 30-bus test system).

Parameter	Without DG	With DG
$P_{loss}^{max}$(MW)	5.8482	5.8482
$P_{loss}^{min}$(MW)	3	2.5
VSEI^*max*^	0.173	0.173
VSEI^*min*^	0.12	0.09
$QC_{RM}^{max}\;(\text{Mvar})$	40	45
$QC_{RM}^{min}\;(\text{Mvar})$	0	0

#### 4.1.1 Single objective optimization

At first, single target optimization is carried out sequentially for each objective function by implementing the proposed FLP algorithm with and without the effect of DG. This issue is important because of several reasons. First, the range of each objective function ($F_{i}^{max}$ and $F_{i}^{min}$) can be identified by decision makers for multi-objective optimization cases as initial and target values of the OPF objectives. Second, this process is useful in investigating conflicts among problem objectives. Finally, this method can be used to validate the outcomes of the DG placement method, study the effect of DG on the solution for the OPF problem for each objective function, and set the control variables. Based on the findings obtained from Eq (42), bus 30 is the most candidate site for DG placement with the highest sensitivity of real power losses to both of the injected real and reactive power which are (-0.1359) and (-0.0477), respectively. Meanwhile, bus 3 is the worst site with the lowest sensitivity of real power losses to both of the injected real and reactive power which are (-0.0391) and (0.006), respectively. In this section, the most candidate site (bus 30) and the worst site (bus 3) are considered for DG placement during the execution of the proposed FLP algorithm with 10 MW maximum capacity and 0.85 p.f. Table 3 shows the optimum settings of the control variables and the values of the objective functions for base case power flow, as well as single objective function optimization cases using the proposed FLP technique without and with DG. A comparison between the results of the proposed FLP algorithm and those of other approaches is presented in Table 4. The following are the studied cases for single objective optimization using the proposed FLP algorithm.

**Table 3:** Single objective optimization using the proposed FLP technique without and with considering DG (IEEE 30-bus test system).

	Limits			Case 1			Case 2		
Item	Min	Max	Base case	Without DG	DG unit at bus 30	DG unit at bus 3	Without DG	DG unit at bus 30	DG unit at bus 3
P_G1_ (MW)	50	200	99.248	51.591	50.0001	50.0274	127.1703	120.9700	120.9335
P_G2_ (MW)	20	80	80	79.997	78.39	78.41	38.8481	37.9305	38.1057
P_G5_ (MW)	15	50	50	49.997	48.67	48.70	44.7625	43.8287	43.9366
P_G8_ (MW)	10	35	20	34.997	33.67	33.65	33.9201	33.10441	33.2935
P_G11_ (MW)	10	30	20	29.997	28.67	28.61	26.7186	25.9582	25.7284
P_G13_ (MW)	12	40	20	39.997	38.64	38.60	17.3973	16.5833	16.6823
V_G1_ (p.u)	0.95	1.1	1.05	1.0573	1.0559	1.0580	1.0726	1.0502	1.0589
V_G2_ (p.u)	0.95	1.1	1.04	1.0537	1.0506	1.0515	1.05139	1.03833	1.04011
V_G5_ (p.u)	0.95	1.1	1.01	1.0338	1.0295	1.0306	1.0618	1.0434	1.0398
V_G8_ (p.u)	0.95	1.1	1.01	1.0423	1.0404	1.0392	1.0429	1.0262	1.0292
V_G11_ (p.u)	0.95	1.1	1.05	1.0623	1.0576	1.0596	1.0996	1.0792	1.0811
V_G13_ (p.u)	0.95	1.1	1.05	1.0591	1.0507	1.0528	1.0532	1.0327	1.0286
T_6,9_	0.9	1.1	1.078	1.007	1.016	1.015	1.0391	1.0517	1.0458
T_6,10_	0.9	1.1	1.069	0.955	0.965	0.964	0.9613	0.9788	0.9704
T_4,12_	0.9	1.1	1.032	1.041	1.047	1.046	0.9956	1.0104	1.0163
T_28,27_	0.9	1.1	1.068	0.983	0.995	0.989	0.984	0.997	0.991
Q_C10_ (Mvar)	0	5	0	1.042	1.949	2.061	4.1518	2.627	3.023
Q_C12_ (Mvar)	0	5	0	4.135	2.190	2.244	4.4172	3.027	2.932
Q_C15_ (Mvar)	0	5	0	4.405	2.036	2.099	4.2275	2.827	2.747
Q_C17_ (Mvar)	0	5	0	1.859	1.939	2.025	4.9934	2.748	2.979
Q_C20_ (Mvar)	0	5	0	4.521	1.922	1.934	4.8539	2.455	3.212
Q_C21_ (Mvar)	0	5	0	4.891	1.872	2.018	4.9392	2.912	2.658
Q_C23_ (Mvar)	0	5	0	4.829	1.856	2.039	4.9482	2.421	2.891
Q_C24_ (Mvar)	0	5	0	4.916	1.666	2.067	4.9289	2.464	3.288
Q_C29_ (Mvar)	0	5	0	4.610	0.840	2.275	4.9352	3.064	2.688
Loss (MW)			5.8482	**3.1797**	**2.6572**	**3.2876**	5.4169	4.9751	5.2800
VSEI			0.173	0.128	0.0961	0.1369	**0.1013**	**0.0978**	**0.1032**
QC_RM_ (Mvar)			45	9.788	28.72	26.23	2.6047	20.45	18.58
P_DG_ (MW)	0	10			8.2270	8.6001		10	10
Q_DG_ (Mvar)		P.F is 0.85			5.0986	5.3299		6.1974	6.1974
Cost ($/h)			902.0207	967.8208	929.5916	929.6592	856.9716	820.3356	821.5810
λ				0.936	0.953	0.764	0.915	0.9506	0.448
CT (S)				6.357	6.567	6.654	6.604	6.948	7.185
No. of iterations				6	6	6	7	7	7

The values in bold type indicate the optimum values.

**Table 4:** Comparison of the proposed FLP technique with different algorithms for real power loss minimization (IEEE 30-bus system).

Method	Loss (MW)
DE [11]	3.24
EGA [12]	3.2008
PSO [12]	3.6294
FLP	3.1797
FLP with DG at bus. 30	2.6572

#### Case 1: Active power losses minimization

**Fig 5:**
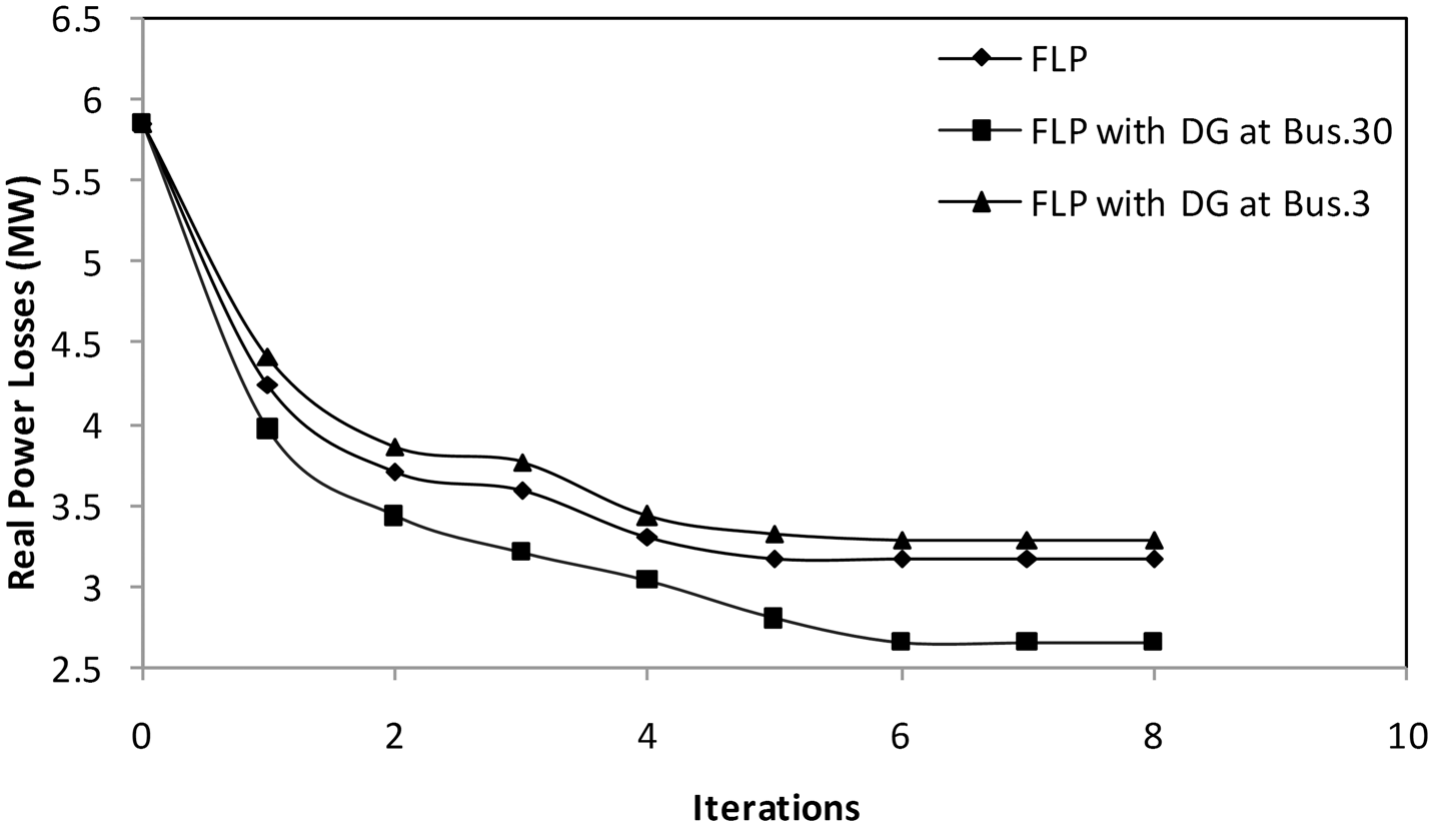
Convergence plot of real power loss minimization using FLP without and with considering DG.

In this case, only active power loss minimization is considered the objective function. Fig 5 shows the convergence of real power loss minimization using the proposed FLP algorithm without and with considering the DG unit at buses 30 and 3consecutively. The proposed technique completely converges to the optimum solution after only six iterations. Furthermore, Table 3 clearly demonstrates the short computational time of the FLP algorithm, which is determined to be 6.357, 6.567, and 6.654 s when the proposed algorithm is employed without DG, with a DG unit at bus 30, and with a DG unit at bus 3, respectively. Such periods are competitive in solving the OPF problem. As shown in Table 3, the proposed FLP algorithm yields a high satisfaction factor for the extreme target objective function value. These results indicate the efficacy of the proposed FLP algorithm in satisfying the optimum solution with a rapid convergence. The results in Table 3 and Fig 5 demonstrate a significant reduction in real power loss from 5.8482 MW to 3.1797 MW in the case where the proposed FLP is implemented without considering DG. In Table 4, the optimal power loss value estimated by the proposed technique is superior to those obtained using EGA [12], PSO [12], and DE [11]. The predetermined target value for the problem objective leads to the fast identification of the global optima region with enhanced exploration capability. Moreover, we notice that accommodating a DG unit at load bus 30, which is determined as the best bus for DG placement by the sensitivity formula, leads to a considerable reduction in power loss (2.6572MW), a low voltage stability index value (0.0961), a considerable saving in generation cost (38.2292$/h), and a significant increment in shunt capacitors MVAR reserve margin from 9.788 MVAR to 28.72 MVAR. This process is accomplished by generating 8.227 MW and 5.0986 MVAR from the DG unit assigned based on the results of the proposed FLP algorithm. By contrast, 8.6001 MW and 5.3299 MVAR, which are the optimum estimated size of the DG unit placed in bus 3 (i.e., the worst defined location for DG placement), produces insignificant loss reduction (3.2876 MW). This value is not even lower than that obtained for loss reduction without considering DG, that is, 3.1797 MW. These findings prove that the erroneous location of DG may increase loss instead of decrease it, and thus, the DG placement method is validated. Based on the results, the proper allocation of DG units, along with the proposed FLP algorithm, optimally minimize active power loss minimization, considerably enhance power system voltage stability, and maximize shunt capacitors MVAR reserve margin, thus leading to a considerable saving in generation cost.

#### Case 2: Voltage stability enhancement

**Fig 6:**
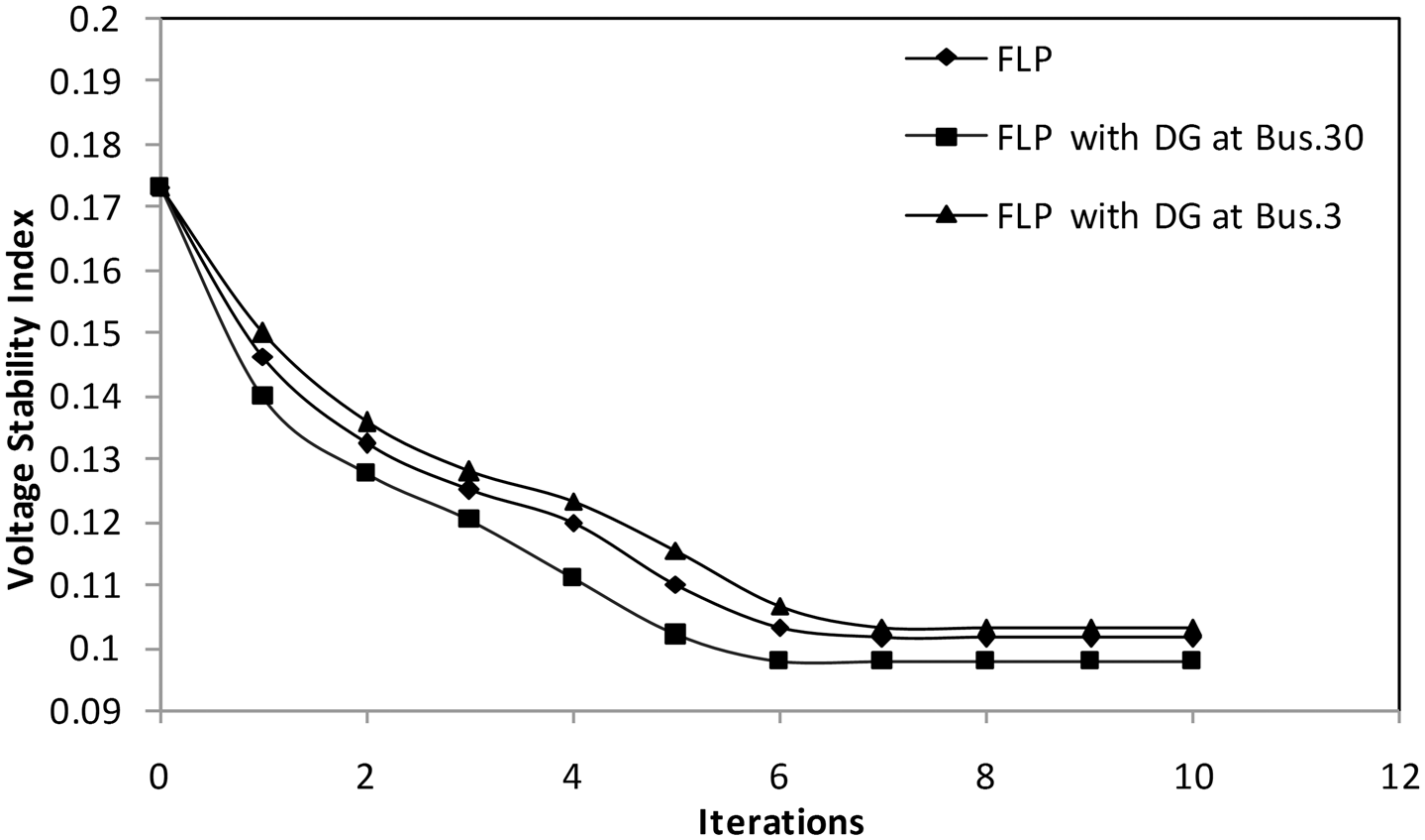
Convergence plot of VSEI using FLP without and with considering DG.

This case considers the minimization of the voltage stability index as an objective function. The convergence plot of the proposed fuzzy algorithm is illustrated in Fig 6 for voltage stability index minimization without and with using a DG unit at buses 30 and 3sequentially. As shown in the Fig 6, the value of the voltage stability index settles at the minimum point (0.1013) after seven iterations, and this value does not change thereafter when the proposed FLP is used without DG. Furthermore, Table 3 shows that optimizing the voltage stability index results in a slight decrease of 7.37% in power loss and a relatively small reservation of shunt compensators reactive power (2.6047 MVAR). Based on Table 3, in the case where a DG unit is accommodated in bus 30, the proposed algorithm converges to a low voltage stability index value (0.0978) and leads to a considerable decrease of 8.155% in power loss compared with the values obtained from the optimizing voltage stability index without considering DG. Notably, a considerable saving in generation cost (36.636$/h) is achieved in this case. As a test location for DG placement, bus 3 produces an increment of 1.875% in the voltage stability index and a minimal reduction of 2.527% in power loss. The results clearly indicate that the optimum allocation of DG leads to a considerable enhancement of system voltage stability. Table 3 also indicates that DG placement improves the MVAR reserve margin of shunt capacitors. When the proposed FLP algorithm is used without considering DG, the execution time is 6.604 s. Meanwhile, when FLP considers a DG unit in buses 30 and 3, computation time slightly increases to 6.948 s and 7.185 s, respectively. Based on the results in Table 3, high satisfaction factors for the extreme targeted values of VSEI minimization are achieved by using the proposed FLP algorithm without and with considering DG at the optimal location, which reflects the reliability of the proposed approach.

#### 4.1.2 Multi-Objective optimization

In this section, three multi-objective optimization combinations are simultaneously solved using the proposed MFLP algorithm. Based on the findings obtained in the previous section, only bus 30 is considered the location for DG after it has been proven to be the best bus for DG accommodation. Similarly, 10 MW maximum capacity and 0.85 p.f are considered for the used DG type. Table 5 presents the optimal value for each objective function, the achieved satisfaction, and the processing time for multi-objective optimization cases using the proposed MFLP algorithm without and with DG effect. A comparison between the obtained results using the proposed MFLP algorithm and other methods is presented in Table 6. On the other hand, emphasis is placed on the comparison between the results obtained using the proposed algorithm without and with considering DG. The objective is to verify the effect of DG on achieving more optimality. In the next subsection, a discussion on the studied cases is presented.

**Table 5:** Multi-Objective optimization using the proposed MFLP technique without and with considering DG(IEEE 30-bus test system).

	Limits			Case 3		Case 4		Case 5	
Item	Min	Max	Base case	Without DG	DG unit at bus 30	Without DG	DG unit at bus 30	Without DG	DG unit at bus 30
P_G1_ (MW)	50	200	99.248	51.9798	50.3995	51.6067	50.1671	51.8653	51.7390
P_G2_ (MW)	20	80	80	79.9934	78.4862	79.9978	78.1285	79.9817	78.0179
P_G5_ (MW)	15	50	50	49.9878	48.4870	49.9991	48.1348	49.9899	48.0925
P_G8_ (MW)	10	35	20	34.9795	33.4270	35	33.3164	34.9746	33.2088
P_G11_ (MW)	10	30	20	29.9783	28.4098	30	29.0487	29.9155	29.1794
P_G13_ (MW)	12	40	20	39.9795	38.2824	39.9958	38.1992	39.9827	38.0833
V_G1_ (p.u)	0.95	1.1	1.05	1.0423	1.0508	1.0688	1.05947	1.0623	1.05716
V_G2_ (p.u)	0.95	1.1	1.04	1.0458	1.0465	1.06099	1.05289	1.05684	1.05114
V_G5_ (p.u)	0.95	1.1	1.01	1.0318	1.0270	1.03098	1.02375	1.03174	1.02404
V_G8_ (p.u)	0.95	1.1	1.01	1.0360	1.0254	1.03785	1.02977	1.03912	1.03022
V_G11_ (p.u)	0.95	1.1	1.05	1.0630	1.0680	1.08669	1.08172	1.08805	1.08212
V_G13_ (p.u)	0.95	1.1	1.05	1.0341	1.0477	1.04482	1.03563	1.04558	1.03604
T_6,9_	0.9	1.1	1.078	0.9979	1.0039	1.0272	1.0332	1.0428	1.0265
T_6,10_	0.9	1.1	1.069	0.9686	0.9721	0.9378	0.9458	0.9203	0.9366
T_4,12_	0.9	1.1	1.032	1.0450	1.0476	0.9854	0.9937	1.0068	1.0152
T_28,27_	0.9	1.1	1.068	0.9807	1.0099	0.975	0.9899	0.9672	0.9964
Q_C10_ (Mvar)	0	5	0	0.4867	0.5247	3.8923	1.378	1.987	1.181
Q_C12_ (Mvar)	0	5	0	0.6319	0.1738	5	2.286	1.309	0.344
Q_C15_ (Mvar)	0	5	0	0.7384	0.7453	4.9105	2.418	0.866	0.938
Q_C17_ (Mvar)	0	5	0	1.2246	0.5279	4.8443	3.371	2.184	0.639
Q_C20_ (Mvar)	0	5	0	0.6561	0.1995	4.9949	2.386	1.286	0.238
Q_C21_ (Mvar)	0	5	0	0.8048	0.5235	4.8288	3.861	0.656	0.742
Q_C23_ (Mvar)	0	5	0	0.6313	0.2099	4.8772	1.891	2.023	0.628
Q_C24_ (Mvar)	0	5	0	0.8944	0.349	3.9112	2.218	0.615	0.355
Q_C29_ (Mvar)	0	5	0	0.6618	0.5664	5	1.981	0.854	0.735
Loss (MW)			5.8482	**3.495**	**3.01**	**3.1994**	**2.544**	**3.298**	**2.904**
VSEI			0.173	0.144	0.1027	**0.1248**	**0.1004**	**0.1061**	**0.1028**
QC_RM_ (Mvar)			45	**38.27**	**41.18**	2.7408	23.21	**33.22**	**39.2**
P_DG_ (MW)	0	10			8.9255		8.422		8.112
Q_DG_ (Mvar)		P.F is 0.85			5.5315		5.2194		5.0273
Cost ($/h)			902.0207	968.4110	925.7502	967.8796	923.1170	967.8142	925.6734
λ				0.8262	0.847	0.90943	0.8746	0.806	0.845
CT (s)				7.378	7.452	7.761	7.8336	7.891	8.049
No. of iterations				6	5	6	7	7	8

Values written in bold type indicate the optimum values.

**Table 6:** Comparison of the proposed MFLP technique with different algorithms for multi-objective optimization cases using the IEEE 30-bus system.

	Case 3				Case 4				Case 5				
Method	Loss (MW)	QC_RM_ (Mvar)	CT (s)	No. of iterations	Loss (MW)	VSEI	CT (s)	No. of iterations	Loss (MW)	QC_RM_ (Mvar)	VSEI	CT (s)	No. of iterations
FPSO [12]	NA	NA	NA	NA	5.5779	0.114	NA	NA	NA	NA	NA	NA	NA
NSGA-II [33]	3.527	37.11	8.75	18	3.2048	0.1250	8.92	22	3.316	32.08	0.1064	9.37	26
NKEA [34]	3.541	35.58	10.33	24	3.2383	0.1254	10.63	30	3.344	31.36	0.1071	11.16	38
DE [35]	3.536	36.08	14.74	36	3.2207	0.1252	15.18	44	3.325	31.48	0.1067	15.32	52
MFLP	3.495	38.27	7.378	6	3.1994	0.1248	7.761	6	3.298	33.22	0.1061	7.891	7
MFLP with DG at bus. 30	3.01	41.18	7.452	5	2.544	0.1004	7.8336	7	2.904	39.2	0.1028	8.049	8

#### Case 3: Simultaneous minimization of real power loss and maximization of shunt capacitors MVAR reserve

**Fig 7:**
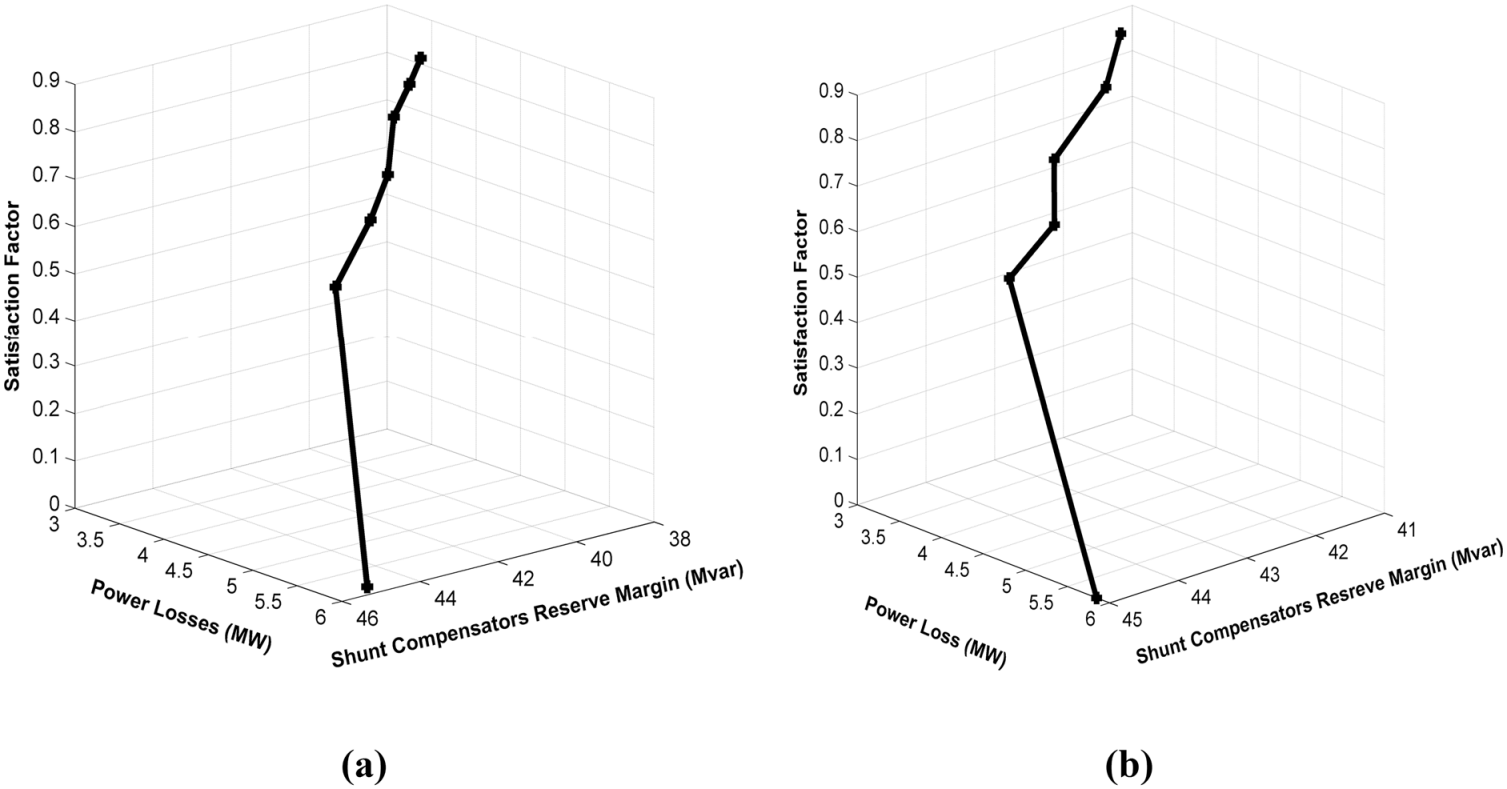
Satisfaction graph of MFLP optimization for loss minimization and shunt compensators MVAR reserve margin maximization. (a) without considering DG; (b) with a DG unit at bus 30.

In this case, two contending objectives, namely, power loss minimization and shunt compensators MVAR reserve margin maximization, are optimized simultaneously using the proposed MFLP technique. Fig 7(a) and 7(b) illustrate the satisfaction plot of the proposed MFLP approach for these objectives without and with considering a DG unit at bus 30, respectively. According to the convergence graphs (Fig 7(a) and 7(b)), the proposed MFLP method results in high satisfaction for the extreme target objective functions values. The proposed technique completely converges to the optimum solution after only six iterations. Consequently, the computational time required to execute MFLP is 7.378 s and 7.452 s without and with DG, respectively. This computational time for solving optimization problems is short. The results exhibit the speed and superiority of the proposed algorithm compared with other heuristics approaches for solving the MOOPF problem, which require dozens, or even hundreds, of iterations. Table 5 presents the values of the objective functions. The optimum solution achieved is 3.495 MW and 38.27 MVAR, which are equivalent to a 40.23% reduction in loss and 85% savings in shunt compensators MVAR injection. Executing the MFLP algorithm for the same objectives while considering the DG unit at bus 30 provides a considerable reduction in loss of 3.01 MW and significant maximization of shunt compensators MVAR reserve margin of up to 41.18 MVAR, that is, savings of 91.5%. Moreover, a considerable saving in generation cost (42.6608$/h) is obtained. The generation of the optimum real and reactive power of the DG unit at bus 30 is estimated by the proposed MFLP to be 8.9255 MW and 5.5315 MVAR, respectively. The achieved satisfaction is excellent considering the assigned extreme target values. For DG, the outcomes shown in Fig 7(b) and Table 5 indicate the positive effect of utilizing DG when solving this case of multi-objective optimization problems. These results reinforce the validity of DG placement. For comparison and further validation, the obtained solution using the proposed MFLP algorithm is compared with those obtained by (NSGA-II) [33], (NKEA) [34], and (DE) [35] algorithms as shown in Table 6. Notably, the results obtained by the proposed MFLP algorithm are attractive results in terms of solution optimality, convergence, and CPU time. In fact, the execution time of the proposed MFLP algorithm is shorter than those of (NSGA-II) [33], (NKEA) [34], and (DE) [35] which are determined to be 8.75, 10.33, and 14.74 s, respectively. Comparing the results listed in Tables 3 and 5, it can be noted that there is a considerable contradiction for multi-objective optimization. For instance, in case of solving OPF without considering DG, the shunt capacitors MVAR reserve increase to 38.27 MVAR but the real power losses increase to 3.495 MW. Eventually, we can confirm that power loss and shunt capacitors MVAR reserve are conflicting objectives based on the results.

#### Case 4: Simultaneous minimization of real power loss and the voltage stability index

**Fig 8:**
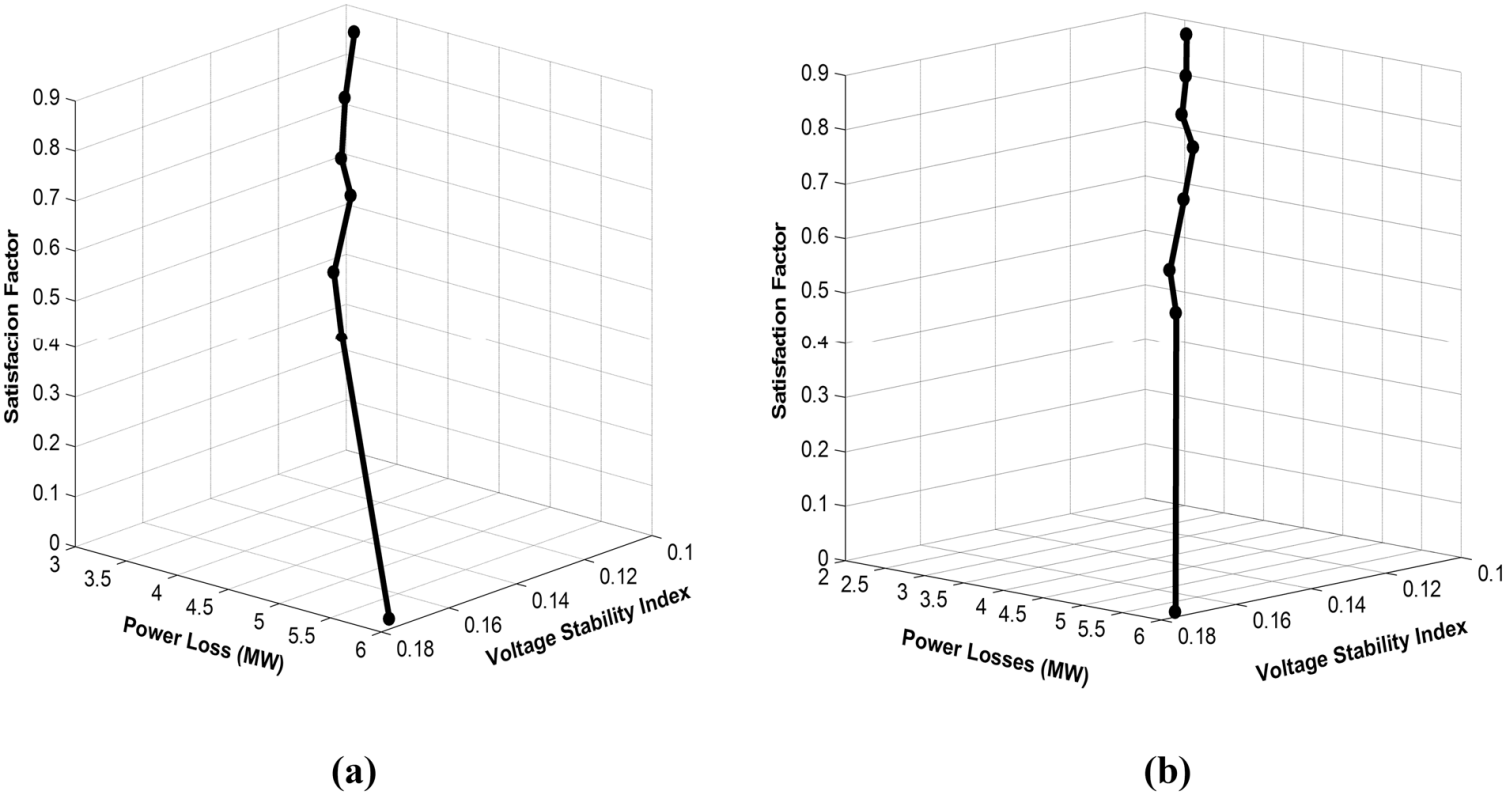
Satisfaction graph of MFLP optimization for loss minimization and voltage stability enhancement. (a) without considering DG; (b) with a DG unit at bus 30.

In this case, two objectives are considered: minimization of real power loss and the voltage stability index. These two objectives are optimized simultaneously using the proposed MFLP algorithm. Fig 8(a) depicts the satisfaction graph of the proposed MFLP technique for these objectives when optimized simultaneously without considering DG. The unique solution obtained is 3.1994 MW and 0.1248 for power loss and the voltage stability index, respectively, with a high satisfaction of 0.90943. In this case, the proposed MFLP algorithm takes six iterations to converge. Meanwhile, Fig 8(b) shows the convergence graph for the same objectives while accommodating a DG unit at bus 30. The best obtained solution is 2.544 MW and 0.1004, with a remarkable satisfaction of 0.8746, which is superior to the obtained solution without DG in Fig 8(a). Comparing the results listed in Tables 3 and 5, the obtained values for power loss and VSEI that are estimated in Table 5 are close to those obtained after individual optimization (Table 3) without and with DG. The results indicate that these objective functions are slightly conflicting. To determine the efficacy of the proposed MFLP algorithm in optimizing loss and voltage stability, a comparison between the optimum solution obtained by the proposed technique and four heuristics algorithms, namely, FPSO [12], NSGA-II [33], NKEA [34], and DE [35] is conducted. The results of this comparison are presented in Table 6, which shows that the proposed MFLP can provide better results than the other algorithms. Moreover, the results exhibit the speed of the proposed algorithm compared with other heuristics approaches for solving the MOOPF problem, which require dozens of iterations. These findings highlight the potential and superiority of the proposed MFLP algorithm over other methods. Lastly, by comparing the obtained results for this case shown in Table 5, the proposed MFLP algorithm that considers DG converges to lower values of objective functions compared with the MFLP algorithm that does not consider DG effect.

#### Case 5: All objectives are optimized simultaneously

**Fig 9:**
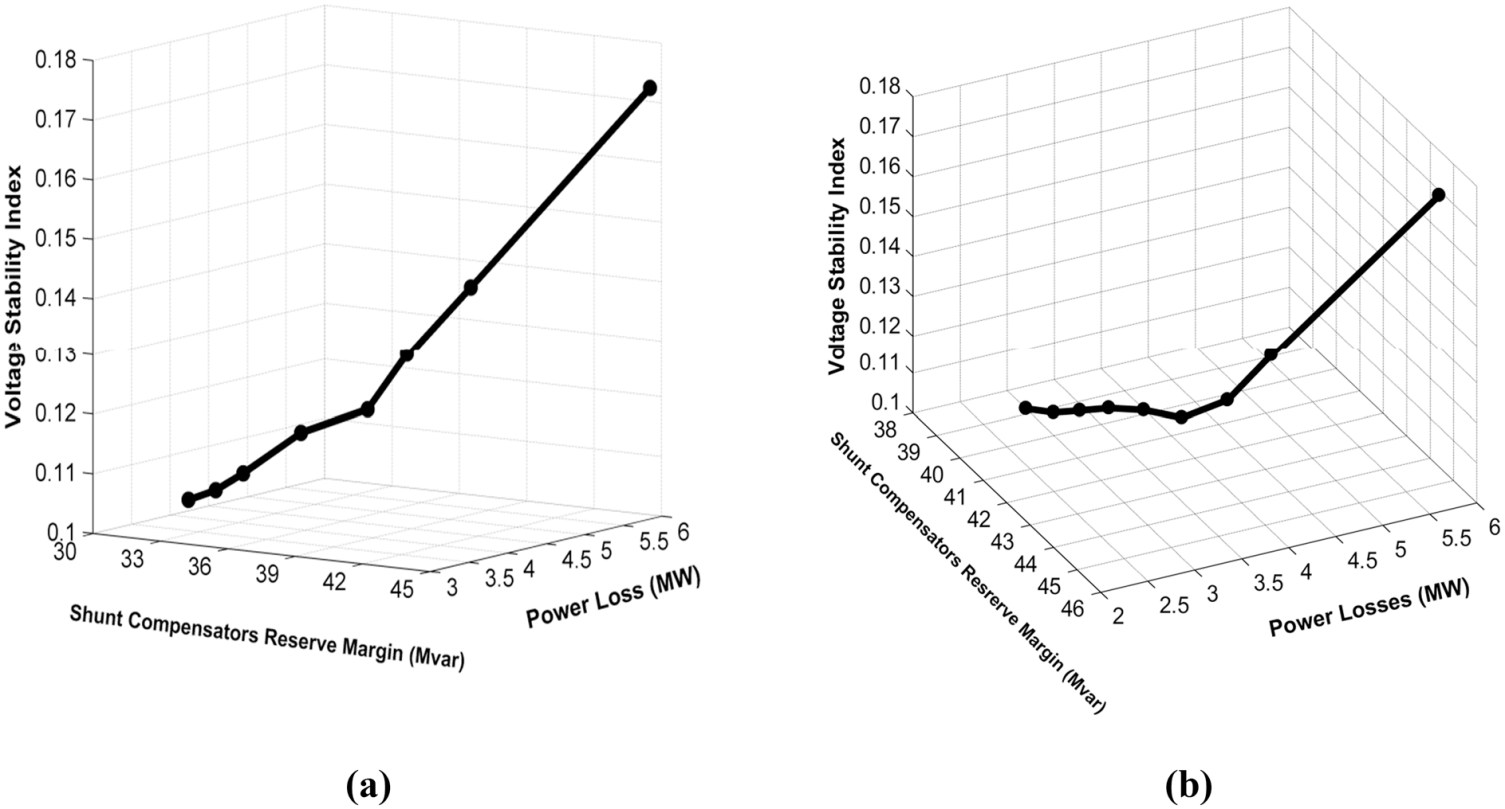
Satisfaction graph of MFLP optimization for the three objective functions. (a) without considering DG; (b) with a DG unit at bus 30.

In the last case, the three selected competing objective functions for the OPF problem are optimized simultaneously by the proposed MFLP algorithm. Fig 9(a) depicts the satisfaction graph for these objectives without considering DG. Seven iterations of the proposed MFLP are adequate to achieve the desired convergence with a computational time of 7.891 s, which is a short execution time for solving multi-objective optimization problems. As listed in Table 5, the optimum solution attained is 3.298 MW, 0.1061, and 33.22 MVAR, with a considerable satisfaction of 0.806 for the target objectives. Meanwhile, Fig 9(b) presents the satisfaction graph for the same objectives by considering a DG unit at bus 30. Marginally different from the previous condition, the proposed MFLP converges after eight iterations, with a computational time of 8.049 s. In Table 5, the satisfaction factor increases to 0.845 when the proposed MFLP algorithm considers the DG effect. The corresponding best solution obtained is 2.904 MW, 0.1028, and 39.2 MVAR. The optimum saving ingeneration cost is determined to be 42.1408 $/h. This solution is achieved parallel with the injection of optimum real and reactive power generation of the allocated DG unit at bus 30, which are 8.112 MW and 5.02736 MVAR, respectively. Thus, Table 5 indicates that the highest quality solution for optimization loss, MVAR reserve, voltage stability, and saving in generation cost is attained by the proposed algorithm when DG is accommodated at the optimum placement compared with without DG. Once more, the efficiency of the proposed MFLP algorithm in solving multi-objective optimization problems is proven. Furthermore, the role of the robustness of the DG placement formula in producing an enviable solution for the MOOPF problem, together with the proposed MFLP algorithm, has been ascertained. Similarly, the optimum solution obtained by the proposed MFLP algorithm is better than those achieved using (NSGA-II) [33], (NKEA) [34], and (DE) [35]. This finding demonstrates the superiority of the suggested algorithm over heuristics methods. Lastly, we can confirm that power loss, voltage stability and shunt capacitors MVAR reserve are contradictory objectives based on the obtained results in Table 5. Notably, all control and dependent variables remain within their permissible constraints.

#### 4.1.3 Statistical analysis

To evaluate the robustness and reliability of the proposed MFLP algorithm in solving the MOOPF problem, a statistical study was conducted. In this work, the MFLP was run 10 times for the three considered multi-objective optimization cases, namely, Case 3,Case 4, and Case 5. The extreme target values for the problem objectives $P_{loss}^{min}$, VSEI^min^, and $QC_{RM}^{max}$ which are displayed in Table 2 are varied extremely and symmetrically with different step sizes as follows:

1. In step size of 0.1 MW, the value of $P_{loss}^{min}$ varies from 3 MW to 2.1 MW and from 2.5 MW to 1.6 MW when solving OPF without and with considering DG, respectively.
2. In step size of 0.01, the values of VSEI^min^ vary from 0.12 to 0.03 and from 0.09 to 0.00 when solving OPF without and with considering DG, respectively.
3. In step size of 1 MVAR, the values of s vary from 40 MVAR to 31 MVAR and from 45 MVAR to 36 MVAR when solving OPF without and with considering DG, respectively.

**Fig 10:**
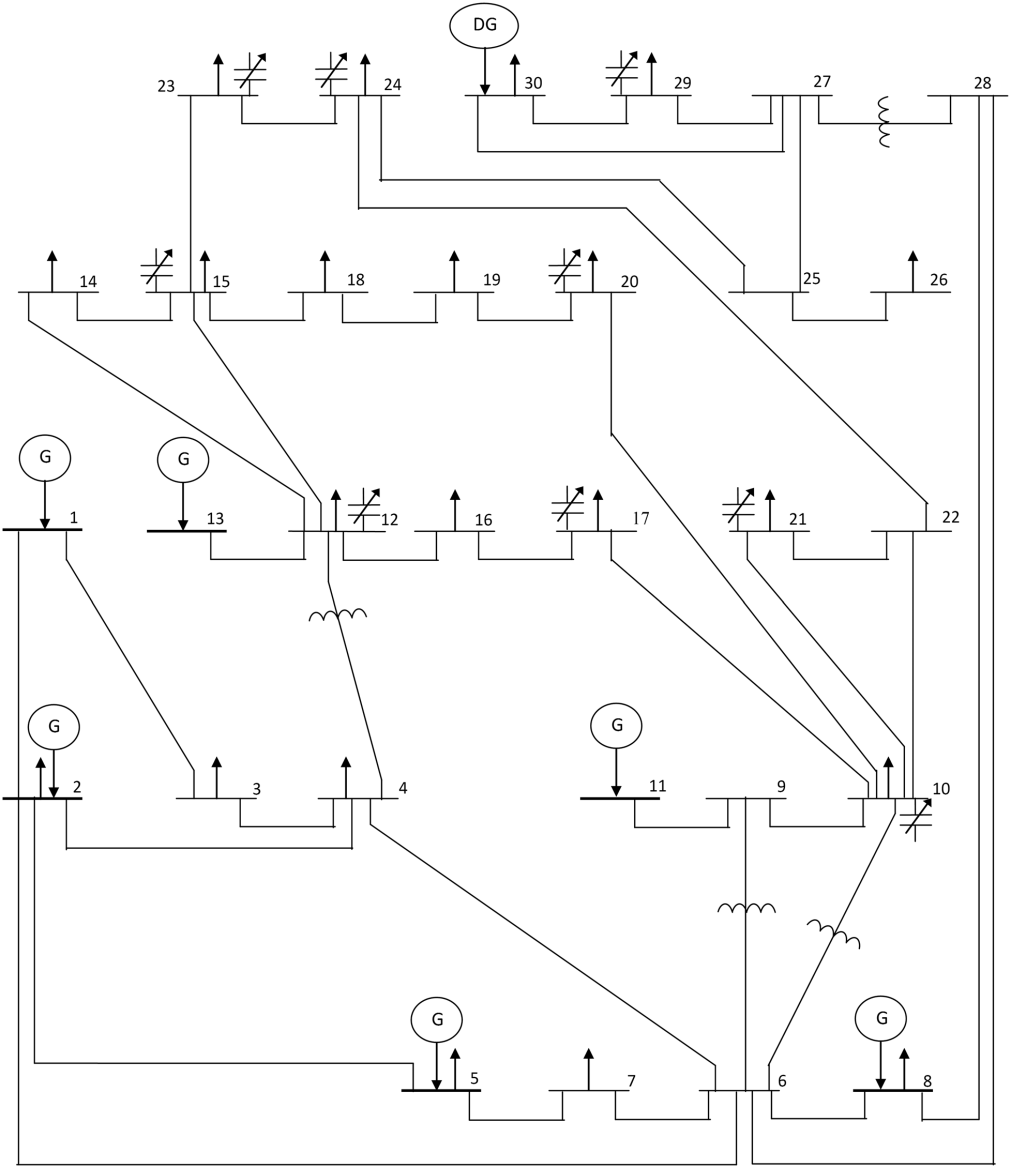
Single line diagram of IEEE 30-bus test system considering the optimal placement of DG.

The used statistical analysis factors for each objective are the best value, the mean value, the worst value, the variance (VR) and the standard deviation (SD). These statistical factors are depicted in Tables 7 and 8 which present the low values of the standard deviation for all considered cases without and with DG, respectively. We notice that the proposed MFLP approach has the ability to settle at the optimum value or very close to it in every run/trial. A gain, this result reveals the effectiveness of the suggested approach in solving optimization problems. Lastly, based on the optimization results (Section 4.1.1 and Section 4.1.2), as well as the statistical analysis results, Fig 10 shows the single line diagram of IEEE 30-bus test system that contains the optimal placement of DG and the locations of reactive power adjustment.

**Table 7:** Statistical results for the proposed MFLP technique without considering DG(IEEE 30-bus test system).

	Case 3		Case 4		Case 5		
Item	Loss (MW)	QC_RM_ (Mvar)	Loss (MW)	VSEI	Loss (MW)	QC_RM_ (Mvar)	VSEI
Best solution	3.495	38.27	3.1994	0.1248	3.298	33.22	0.1061
Worst solution	3.503	37.71	3.2033	0.125	3.312	33.05	0.1083
Mean	3.498	38.04	3.1999	0.12485	3.299	33.19	0.1064
Variance	0.00018	0.0021	0.00013	0.0015	0.00005	0.0014	0.00071
Standard deviation	0.01341	0.0458	0.0114	0.0387	0.00707	0.03741	0.02664

**Table 8:** Statistical results for the proposed MFLP technique with considering DG(IEEE 30-bus test system).

	Case 3		Case 4		Case 5		
Item	Loss (MW)	QC_RM_ (Mvar)	Loss (MW)	VSEI	Loss (MW)	QC_RM_ (Mvar)	VSEI
Best solution	3.01	41.18	2.544	0.1004	2.904	39.2	0.1028
Worst solution	3.08	41.01	2.583	0.1011	2.914	39.04	0.1033
Mean	3.02	41.15	2.552	0.1007	2.909	39.11	0.103
Variance	0.00012	0.0032	0.00009	0.001	0.000041	0.0026	0.00037
Standard deviation	0.01095	0.0565	0.00948	0.03162	0.006403	0.05099	0.01923

### 4.2 IEEE 118-bus test system

In order to prove the effectiveness and scalability of the proposed MFLP procedure for large-scale power networks, a standard IEEE 118-bus test network was utilized. The complete data of this system including branch parameter, load data, generator data, and the initial setting of the control variables with their corresponding operational constraints can be found in [37]. This system consists of 186 transmission lines, 54 generator buses, and 64 load buses. Nine branches, namely, (8–5), (26–25), (30–17),(38–37),(63–59),(64–61),(65–66),(68–69), and (81–80), are equipped with tap-changing transformers. Meanwhile, load buses 34, 44, 45, 46, 48, 74, 79, 82,83,105,107, and 110 are chosen as locations for the switchable shunt capacitors. On the one hand, the minimum and upmost boundaries of the voltage magnitude of all buses are set to [0.95, 1.1] p.u. On the other hand, the upper and lower limits for each transformer tap are 0.9 p.u and 1.1 p.u, respectively. The MVAR injected by each capacitor bank is within 0–30 MVAR. Table 9 presents the assigned parameters of the fuzzy membership functions for the IEEE 118-bus test system.

**Table 9:** Parameters of the fuzzy membership functions for the problem objectives (IEEE 118-bus test system).

Parameter	Without DG	With DG
$P_{loss}^{max}$(MW)	132.79	132.79
$P_{loss}^{min}$(MW)	110	108
VSEI^*max*^	0.2106	0.2106
VSEI^*min*^	0.11	0.08
$QC_{RM}^{max}\;(\text{Mvar})$	300	360
$QC_{RM}^{min}\;(\text{Mvar})$	0	0

#### 4.2.1 Single objective optimization

Similarly, single target optimization is performed sequentially for the same objective functions considered in Case 1 and Case 2 by implementing the proposed FLP approach with and without the impact of DG. Based on the findings obtained from Eq (42), bus 106 is the most candidate site for DG placement with the highest sensitivity of real power losses to both of the injected real and reactive power which are (-0.1162) and (-0.0588), respectively. Meanwhile, bus 38 is the worst site with the lowest sensitivity of real power losses to both of the injected real and reactive power which are (-0.0039) and (-0.0001), respectively. In this section, the most candidate site (bus 106) is considered for DG placement during the execution of the proposed FLP algorithm using two DG units with 10 MW maximum capacity and 0.85 p.f. Table 10 shows the values of the objective functions for base case power flow, as well as the test results obtained for single objective function optimization cases using the proposed FLP technique without and with DG. Furthermore, a comparison between the results of the proposed FLP algorithm and three heuristics algorithms, namely, (NSGA-II) [33],(NKEA) [34], and (DE) [35] is presented in Table 11. It can be noted that the suggested MFLP method produces superior results in comparison with the above- mentioned algorithms. This confirms that the suggested MFLP method can be used effectively to solve single objective OPF problems for large-scale power networks.

**Table 10:** Single objective optimization using the proposed MFLP technique without and with considering DG(IEEE 118-bus test system).

		Case 1		Case 2	
Item	Base case	Without DG	DG unit at bus. 106	Without DG	DG unit at bus. 106
Loss (MW)	132.79	**112.705**	**109.472**	127.649	124.583
VSEI	0.2106	0.1751	0.1472	**0.1299**	**0.1004**
QC_RM_ (Mvar)	360	98.11	136.82	32.459	111.485
P_DG_ (MW)			17.638		20
Q_DG_ (Mvar)			10.9309		12.3948
λ		0.8813	0.9406	0.8021	0.8437
CT (S)		14.392	14.552	14.704	14.881
No. of iterations		10	10	11	12

Values written in bold type indicate the optimum values.

**Table 11:** Comparison of the proposed FLP technique with different algorithms for single objective optimization cases using the IEEE 118-bus system.

	Single objective	
Method	Loss (MW)	VSEI
NSGA-II [33]	113.579	0.1385
NKEA [34]	114.118	0.1419
DE [35]	113.937	0.1408
FLP	112.705	0.1299
FLP with DG at bus. 106	109.472	0.1004

#### 4.2.2 Multi-Objective optimization

For an extensive validation of the proposed MFLP algorithm, the same three multi-objective optimization combinations as used in 30-bus test network are considered. Similarly, bus 106 is selected as a location for DG placement. Table 12 expresses the optimal value for each objective function, the achieved satisfaction, and the processing time for multi-objective optimization cases using the proposed MFLP algorithm without and with DG effect. Notably, the proposed MFLP algorithm that considers DG achieves more optimal solution compared with the MFLP algorithm that does not consider the DG effect. Comparing the results listed in Tables 10 and 12, a considerable contradiction for multi-objective optimization is noted. Furthermore, a comparison between the obtained results using the proposed MFLP algorithm and the same competitors is presented in Table 13. Obviously, the obtained results by the proposed MFLP are attractive in terms of solution superiority, convergence, and CPU time. Notably, the results demonstrate the speed of the proposed MFLP algorithm compared with other heuristics approaches for solving the MOOPF problem, which require dozens, or even hundreds, of iterations. This proves its efficacy, applicability, and potential in solving multi-objective OPF problems for large-scale power networks.

**Table 12:** Multi-Objective optimization using the proposed MFLP technique without and with considering DG(IEEE 118-bus test system).

		Case 3		Case 4		Case 5	
Item	Base case	Without DG	DG unit at bus 106	Without DG	DG unit at bus 106	Without DG	DG unit at bus 106
Loss (MW)	132.79	**114.326**	**110.829**	**112.119**	**109.022**	**113.881**	**110.133**
VSEI	0.2106	0.1938	0.1163	**0.1288**	**0.0951**	**0.1188**	**0.0994**
QC_RM_ (Mvar)	360	**291.18**	**331.62**	168.11	188.39	**269.76**	**317.85**
P_DG_ (MW)			18.117		18.882		19.198
Q_DG_ (Mvar)			11.227		11.701		11.897
λ		0.8101	0.8858	0.8131	0.8843	0.8297	0.8514
CT (S)		15.583	15.648	15.751	15.817	15.984	16.212
No. of iterations		13	15	13	13	14	16

Values written in bold type indicate the optimum values.

**Table 13:** Comparison of the proposed MFLP technique with different algorithms for multi-objective optimization cases using the IEEE 118-bus system.

	Case 3				Case 4				Case 5				
Method	Loss (MW)	QC_RM_ (Mvar)	CT (s)	No. of iterations	Loss (MW)	VSEI	CT (s)	No. of iterations	Loss (MW)	QC_RM_ (Mvar)	VSEI	CT (s)	No. of iterations
NSGA-II [33]	114.818	288.44	18.28	42	112.771	0.1296	19.03	49	114.205	266.54	0.1196	19.73	55
NKEA [34]	115.365	279.29	19.47	64	113.228	0.1317	19.86	70	114.816	259.21	0.1221	20.95	78
DE [35]	114.992	282.05	26.24	105	113.053	0.1308	28.41	122	114.399	262.74	0.1204	30.27	134
MFLP	114.326	291.18	15.58	13	112.119	0.1288	15.751	13	113.881	269.76	0.1188	15.98	14
MFLP with DG at bus. 106	110.829	331.62	15.64	15	109.022	0.0951	15.817	13	110.133	317.85	0.0994	16.21	16

## 5 Conclusions

This study proposes an efficient MFLP approach to solve the MOOPF problem in mesh-connected power systems without and with considering the effect of DG. Simultaneously, three objectives, namely, power loss minimization, voltage stability enhancement, and shunt compensators MVAR margin maximization, are considered for optimization. The optimum placement of DG units is identified using a sensitivity based-method. The results indicate the competence of the used technique. Meanwhile, the task of finding the optimal DG size is performed by the proposed MFLP approach by considering the generated real power of DG as control variable. The proposed MFLP technique has been scrutinized and validated using the IEEE 30-bus and IEEE 118-bus test systems. The results indicate that a unique and optimum solution with an excellent satisfaction for the targets of the decision makers can be achieved within a short computational time by the proposed MFLP algorithm despite the extreme assigned targets without and with considering DG. The predetermined target value for the problem objective leads to the fast identification of the global optima region with enhanced exploration capability. The findings illustrate the efficacy and reliability of the proposed MFLP technique. In addition, compared with the results in literature, the proposed technique outperforms other heuristics algorithms in terms of solution optimality in several cases of single/multiple objective optimization. Employing the MFLP technique with the proper placement of DG results in higher optimality for the values of problem objectives compared with performing it without considering DG. DG extensively affects power loss minimization and shunt capacitors MVAR reserve margin maximization. Furthermore, a considerable saving in generation cost is achieved. In conclusion, the results show significant and desirable loss reduction, MVAR reserve, and improved voltage stability margin by using the proposed MFLP algorithm without and with considering DG. The proposed approach is convenient for online implementation in real power system operation in term of high satisfaction for the assigned targets of decision makers, distinct convergence property, high exploration capability, and rigid enforcement of system constraints.
